# Characterization and structural analyses of a novel glycosyltransferase acting on the β-1,2-glucosidic linkages

**DOI:** 10.1016/j.jbc.2022.101606

**Published:** 2022-01-19

**Authors:** Kaito Kobayashi, Hisaka Shimizu, Nobukiyo Tanaka, Kouji Kuramochi, Hiroyuki Nakai, Masahiro Nakajima, Hayao Taguchi

**Affiliations:** 1 The Department of Applied Biological Science, Faculty of Science and Technology, Tokyo University of Science, Chiba, Japan; 2 The Faculty of Agriculture, Niigata University, Niigata, Japan

**Keywords:** β-1,2-glucan, β-1,2-glucooligosaccharide, glycosyltransferase, glycoside hydrolase, carbohydrate, structure–function, enzyme, β-galactosidase, β-glucosaminidase, β-1,2-glucanase, α-GlcF, α-d-glucosyl fluoride, CAZy, Carbohydrate-Active enZYmes, Cel_n_ (n is DP), cellooligosaccharide, DNJ, 1-deoxynojirimycin, DP, degree of polymerization, ESI-MS, electrospray ionization-mass spectrometry, Gal, galactopyranoside, GH, glycoside hydrolase family, Glc, glucose, GlcN, glucosamine, GlmA, β-glucosaminindases, IaSGT, IALB_1185 from *Ignavibacterium album* (“S” is derived from sophorooligosaccharides, an alternative name of β-1,2-glucooligosaccharides and “GT” is derived from glucosyltransferase), Lam_n_ (n is DP), laminarioligosaccharide, pNP, *p*-nitrophenyl, SGL, endo-β-1,2-glucanase, SOGP, 1,2-β-oligoglucan phosphorylase, Sop_n_, β-1,2-glucooligosaccharide (n is DP), SpBgaC, β-galactosidase from *Streptococcus pneumoniae* TIGR4, TkGlmA, GlmA from *Thermococcus kodakaraensis*, WT, wild-type

## Abstract

The IALB_1185 protein, which is encoded in the gene cluster for endo-β-1,2-glucanase homologs in the genome of *Ignavibacterium album*, is a glycoside hydrolase family (GH) 35 protein. However, most known GH35 enzymes are β-galactosidases, which is inconsistent with the components of this gene cluster. Thus, IALB_1185 is expected to possess novel enzymatic properties. Here, we showed using recombinant IALB_1185 that this protein has glycosyltransferase activity toward β-1,2-glucooligosaccharides, and that the kinetic parameters for β-1,2-glucooligosaccharides are not within the ranges for general GH enzymes. When various aryl- and alkyl-glucosides were used as acceptors, glycosyltransfer products derived from these acceptors were subsequently detected. Kinetic analysis further revealed that the enzyme has wide aglycone specificity regardless of the anomer, and that the β-1,2-linked glucose dimer sophorose is an appropriate donor. In the complex of wild-type IALB_1185 with sophorose, the electron density of sophorose was clearly observed at subsites −1 and +1, whereas in the E343Q mutant–sophorose complex, the electron density of sophorose was clearly observed at subsites +1 and +2. This observation suggests that binding at subsites −1 and +2 competes through Glu102, which is consistent with the preference for sophorose as a donor and unsuitability of β-1,2-glucooligosaccharides as acceptors. A pliable hydrophobic pocket that can accommodate various aglycone moieties was also observed in the complex structures with various glucosides. Overall, our biochemical and structural data are indicative of a novel enzymatic reaction. We propose that IALB_1185 be redefined β-1,2-glucooligosaccharide:d-glucoside β-d-glucosyltransferase as a systematic name and β-1,2-glucosyltransferase as an accepted name.

Carbohydrate chains are important polymer compounds for all organisms, which is attributed to the wide variety of carbohydrate chain structures. Such complexity of the structures is thought to be responsible for the repertoire of enzymes that synthesize and degrade carbohydrate chains. The functions and structures of these enzymes have become extensively diversified through molecular evolution. To date, various kinds of enzymes related to carbohydrates have been found and added to the Carbohydrate-Active enZYmes (CAZy) database (http://www.cazy.org) (1, 2). This database classifies these enzymes called CAZymes into families mainly based on their amino acid sequences and is now expanding. However, obtaining carbohydrates is often difficult due to their rarity or inhomogeneity in nature, which limits exploration of novel enzymes.

β-1,2-Glucan is a homopolymer composed of β-1,2-linked glucose units and is one of the carbohydrates difficult to obtain from natural resources practically. β-1,2-Glucans are found mainly as cyclic forms or some as linear forms in nature. Some Gram-negative bacteria, such as *Rhizobium* (including species named *Agrobacterium* formerly), *Sinorhizobium*, and *Brucella*, produce cyclic β-1,2-glucans (3, 4, 5). *Chlorella pyrenoidosa*, a unicellular green alga, produces both cyclic and linear forms (6). *Escherichia coli* and *Pseudomonas syringae* produce short linear β-1,2-glucan chains with side chains (7, 8), though they are rare examples of short chains. β-1,2-Glucans are involved in bacterial infection of plant and animal cells, hypo-osmotic adaptation, and iron storage (9, 10, 11). β-1,2-Glucans in nature exhibit some variety in the degree of polymerization (DP). *C. pyrenoidosa*, *Brucella abortus*, and *Rhizobium radiobacter* produce cyclic β-1,2-glucans with DPs of up to 20 (5, 12, 13). Cyclic β-1,2-glucans with DPs of around 40 produced by *Rhizobium meliloti* have also been found (14). DPs of β-1,2-glucans from *E. coli* and *P. syringae* are around 10 (7, 8). Glycosides possessing β-1,2-glucooligosaccharides (Sop_n_s, n is DP) as glycones have been found in plants such as stevia, citrus fruits, and so on (15, 16). Sophorolipids are found in many yeast species (17), and ones from *Candida bombicola* are commercially manufactured (18). β-1,2-Glucans are uniquely quite soluble in both cyclic and linear forms, unlike glucose polymers such as cellulose (β-1,4-glucans) and β-1,3-glucans (19). Cyclic β-1,2-glucans have been reported as inclusion compounds that raise the solubility of hydrophobic molecules (19). However, the physical and physiological functions of β-1,2-glucans have not been investigated sufficiently, which might be related to less progress in exploration of β-1,2-glucan-associated enzymes.

Genes encoding β-1,2-glucan-degrading enzymes had not been identified until a novel phosphorylase was first found in *Listeria innocua* as an enzyme that can act on linear β-1,2-glucans (Hereafter, β-1,2-glucan represents a linear form unless otherwise noted.) in 2014 (20, 21, 22). This enzyme was named 1,2-β-oligoglucan phosphorylase (SOGP) and was given a new EC number (EC2.4.1.333) (23). After that, a putative glycoside hydrolase family (GH) 3 enzyme in the SOGP gene cluster was found to be a β-glucosidase preferably hydrolyzing Sop_2_ by functional and structural analyses (24). A carbohydrate-binding subunit of a putative ABC transporter in the same gene cluster was also found to be a Sop_n_s-binding protein (25). These results are the first biochemical evidence of the existence of a gene cluster involved in β-1,2-glucan metabolism. A large-scale preparation method for β-1,2-glucan has been established using SOGP and inexpensive sugars as materials (26, 27). The prepared β-1,2-glucan was used for identification of endo-β-1,2-glucanases (SGLs) from a bacterium and a fungus. Both SGLs have been successfully identified and classified into new families (GH144 and GH162, respectively) (28, 29). This finding enables us to explore SGL homologs and SGL gene clusters. An SGL homolog possessing an unknown function region at the N terminus has been identified as a novel exolytic enzyme that releases Sop_2_ from the nonreducing ends of Sop_n_s (30). A β-glucosidase preferably acting on longer Sop_n_s and β-1,2-glucan has also been found from an SGL gene cluster in *Bacteroides thetaiotaomicron* (31). The structure–function relationships of these enzymes have also been analyzed (24, 25, 28, 29, 32). However, the abovementioned reports are most of the studies on β-1,2-glucan-degrading enzymes, implying insufficient understanding of the variety of β-1,2-glucan-associated enzymes.

Here, we focus on an SGL gene cluster in the genome from *Ignavibacterium album*, a moderately thermophilic anaerobic bacterium found in a hot spring in Japan (33). This gene cluster includes two genes encoding putative GH144 enzymes, and β-1,2-glucan-related genes encoding a putative GH3 β-glucosidase, a putative GH94 enzyme (a homolog of the SOGP), and a putative Sop_n_s-binding protein in an ABC transporter (Fig. S1). The gene cluster also contains a gene (*ialb_1185*) encoding a putative GH35 enzyme (IALB_1185, hereafter IaSGT). While GH35 enzymes, which are distributed in a wide range of microorganisms, plants, and animals, are mainly β-galactosidases (β-galactosidase, exo-β-1,4-galactanase, and β-1,3-galactosidase) according to the CAZy database (34, 35, 36), several GH35 enzymes from Archaea have been found to be β-glucosaminindases (GlmAs), and GlmA from *Thermococcus kodakaraensis* (TkGlmA) hydrolyzes chitosan and chitooligosaccharides (37, 38). The binding modes of natural substrates are not sufficiently understood, since only the glucosamine (GlcN) complex is available among GlmAs (39). Though many GH35 enzymes have been reported, as described above, no glucoside-acting enzyme has been reported in this family. In this study, we report the first β-1,2-glucan-associated GH35 enzyme biochemically and structurally and furthermore describe why the enzyme is a novel enzyme that should be given a new EC number.

## Results

### Phylogenetic and sequence analysis of IaSGT

Phylogenetic analysis was performed using the amino acid sequences of characterized GH35 enzymes in the CAZy database and IaSGT. While Eukaryotic GH35 enzymes are divided into two clusters, each bacterial and archaeal GH35 enzymes form one cluster, respectively (Fig. S2 and Table S1). Notably, IaSGT and its homologs form a distinct cluster from the known GH35 enzymes. Though the group of archaeal GlmAs is close to that of IaSGT in the phylogenetic tree, the amino acid sequence identity between these enzymes and IaSGT is low (only ∼28%).

IaSGT has no N-terminal signal peptide, suggesting that it is localized in the cytosol. Nucleophile and acid/base residues in GlmAs (Glu347 and Glu179 in TkGlmA, respectively) are conserved in IaSGT (E343 and E176, respectively) (Fig. S3). Though most substrate recognition residues at subsite −1 in the GlmAs (Tyr53, Glu103, Glu179, Glu347, and Tyr379 in TkGlmA) are also conserved in IaSGT, aspartate residues (Asp178 in TkGlmA) considered to be responsible for specificity to the amino group in GlcN are replaced by an asparagine residue in IaSGT (Asn175). Furthermore, subsite plus side regions (around Glu184 and Leu282 in TkGlmA) are not conserved at all. These differences imply that IaSGT has different substrate specificity from the GlmAs.

### General properties

The purified IaSGT migrated as a single band corresponding to approximately 75 kDa/m on an SDS-PAGE gel (Fig. S4*A*). The enzyme was eluted at the time corresponding to 141 kDa/m on size-exclusion chromatography (Fig. S4*B*). Thus, this enzyme should form a dimer. Since IaSGT acted on Sop_2_ to release glucose (Glc) as described below in detail, quantification of Glc was used for investigation of pH and temperature profiles. IaSGT showed high activity at pH 5.0 − 8.0 (over 90% relative activity as to the highest) and was stable at pH 5.0 to 11.0 (Fig. S5*A*). IaSGT showed optimum activity at 55 °C and was stable up to 60 °C after incubation for 1 h (Fig. S5*B*), which is consistent with the bacterial growth property as to temperature.

### Substrate specificity and reaction mode of IaSGT

**Figure 1:**
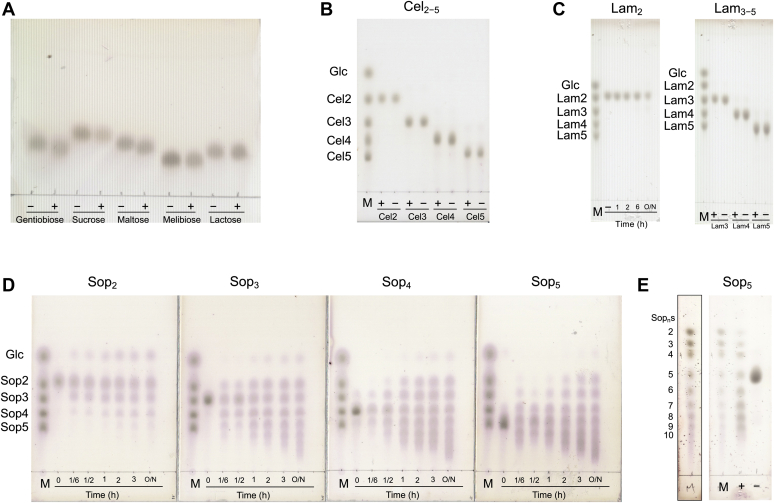
**TLC analysis of activity toward oligosaccharides.** *A–E,* + and − represent with and without WT IaSGT in the reaction mixture, respectively. Substrates are shown above or below the plates. Lane M, markers including 0.5% Glc and Cel_2–5_, Lam_2–5_ or Sop_2–5_. (*C left* and *D*) The reaction times are shown at the *bottom* of the plates. Lane M, the preparation method of the marker is described in the Experimental procedures. Lane M with a different contrast was added as the boxed lane to show the positions of Sop_n_s clearly.

Since most GH35 enzymes show β-galactosidase activity, the activity of IaSGT toward β-galactosides was investigated. However, IaSGT did not show any hydrolytic activity toward lactose (β-Gal-1,4-Glc) (Fig. 1*A*) or *p*-nitrophenyl (pNP)-β-galactopyranoside (Gal), an artificial substrate (less than 0.01 U/mg for 1 mM pNP-β-Gal). Nor did the enzyme act on various oligosaccharides such as cellooligosaccharides (Cel_2−5_), laminarioligosaccharides (Lam_2−5_), maltose (α-Glc-1,4-Glc), gentiobiose (β-Glc-1,6-Glc), or sucrose (α-Glc-1,2-β-Fru) (Fig. 1, *A*–*C*). On the other hand, IaSGT showed activity toward Sop_2−5_ obviously (Fig. 1*D*). IaSGT produced oligosaccharides with both lower and higher DPs than those of the substrates. This disproportionation of DPs proceeded by transfer of a glucose unit. The products appeared not to show decreases in their average DPs even after reaction overnight. The enzyme did not show hydrolytic (glucose-releasing) activity toward Sop_3_ (less than 0.01 U/mg for 20 mM Sop_3_). These results indicated that the reaction mode of IaSGT was that of a glycosyltransferase. After the products at the beginning of the reaction with Sop_3_ were fractionated by size-exclusion chromatography, only the product at the position corresponding to Sop_4_ on the TLC plate was collected and analyzed by ^1^H-NMR (Fig. S6*A*). The chemical shifts of the product fitted completely with those of the reference Sop_4_ (Fig. S6, *B* and *C*), indicating that the reaction product is Sop_4_ and that the enzyme transfers a glucose unit to produce a β-1,2-glucosidic bond. Such elongation by transfer of Glc units has been found in GH16 elongating β-transglycosylase, though the GH16 enzyme acts on β-1,3/1,4-linkages (40). To understand the DPs of the reaction products in detail, the products after the overnight reaction with Sop_5_ were separated on the TLC plate by developing twice. As a result, Sop_n_s with DP at least up to 9 were clearly detected (Fig. 1*E*). This is consistent with the fact that Sop_2−9_ were clearly detected by electrospray ionization–mass spectrometry (ESI-MS) (Fig. S7*A*). Sop_n_s with DPs of 10 or more could also be assigned. Though velocities of disproportionation appeared to slow down after 3 h of the reactions (Fig. 1*D*), this is probably because the proportion of Sop_n_s molecules with the highest and the lowest DPs to all the substrate molecules in the reaction solution was reduced.

### Kinetics for Sop_n_s

In order to determine whether Sop_n_s are appropriate substrates for IaSGT, the kinetic parameters of the glucosyl transferase activity of the enzyme toward Sop_2−5_ were determined (Table 1). Though IaSGT showed modest *k*_cat_ values, the *K*_m_ values were remarkably large, especially for Sop_2_ and Sop_3_ (120 mM and 300 mM, respectively), as a GH enzyme. Consequently, the *k*_cat_/*K*_m_ values were quite small (less than 0.1 s^−1^ mM^−1^ for Sop_2_, Sop_3,_ and Sop_5_, and less than 0.5 s^−1^ mM^−1^ for Sop_4_). Since the substrates in the transferase reaction are both donors and acceptors, the quite large *K*_m_ values suggest that Sop_n_s are inappropriate as at least either donors or acceptors.

**Table 1:** Kinetic parameters of IaSGT for Sop_n_s

Substrate	*k*_cat_ (s^−1^)	*K*_m_ (mM)	*k*_cat_/*K*_m_ (s^−1^ mM^−1^)
Sop_2_	8.0 ± 0.9	120 ± 20	0.069 ± 0.002
Sop_3_	18 ± 1	300 ± 20	0.059 ± 0.001
Sop_4_	4.8 ± 0.2	15 ± 2	0.32 ± 0.02
Sop_5_	1.7 ± 0.2	22 ± 3	0.078 ± 0.005

Concentrations used were 5 to 40 mM, except for Sop_3_ (5–80 mM). All experiments were carried out in triplicate. Standard errors are used in the table.

### Determination of acceptor substrates

**Figure 2:**
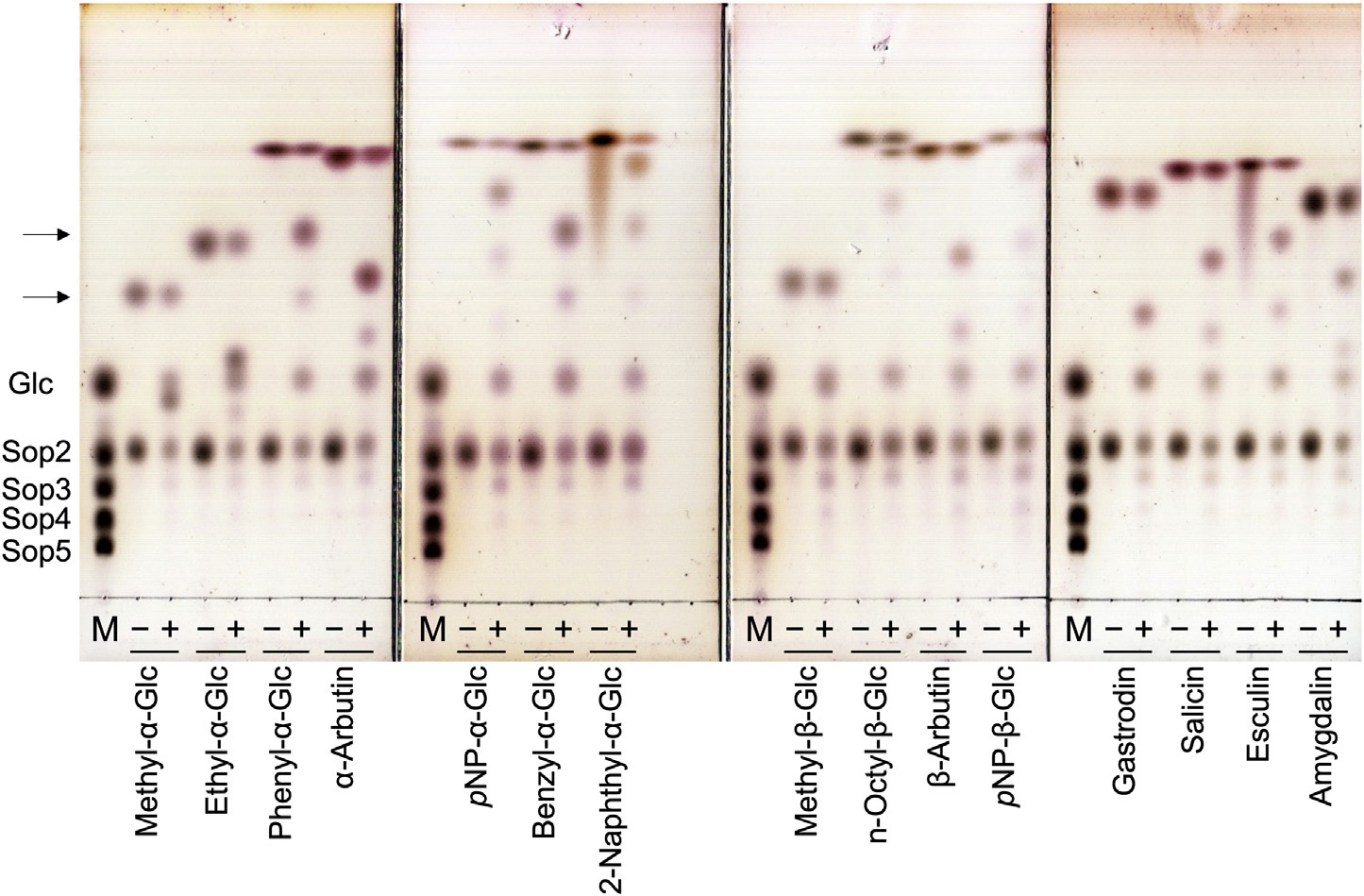
**TLC analysis of glycosyltransfer activity toward various acceptor glucosides.** M, markers containing 0.5% each of Glc and Sop_2−5_; + and − represent with and without WT IaSGT in the reaction mixture, respectively. Acceptors are shown below the TLC plates. *Arrows* indicate the products derived from phenyl-α-Glc. The TLC plates were developed once with 80% (v/v) acetonitrile in water.

To determine optimal acceptors of IaSGT, the effects of various monosaccharides and disaccharides (1 mM d-mannose, d-glucose, d-galactose, d-xylose, d-talose, l-arabinose, d-fructose, l-rhamnose, d-gluconate, Lam_2_, Cel_2_, gentiobiose, sucrose, maltose, α,α-trehalose, or lactose) as acceptors on activity toward 2 mM Sop_2_ were investigated. However, a remarkable increase in specific activity was not found (less than 15% increase in specific activity, data not shown). Then, the glycosynthase activity of the E343G mutant in the presence of α-d-glucosyl fluoride (α-GlcF) as a donor was investigated by TLC analysis. The mutant showed glycosynthase activity only in the presence of glucose as an acceptor among the examined monosaccharides and disaccharides, though α-GlcF itself acted as an acceptor as well (Fig. S8). When pNP-α-Glc was used as an acceptor, a synthetic product was observed. Therefore, various aryl- and alkyl-glucosides, as acceptors, were investigated using the WT IaSGT in the presence of Sop_2_ as a donor. Reaction products were detected regardless of the anomer of acceptors except methyl-β-Glc (Fig. 2). Considering the kinetic analysis described later, spots of reaction products derived from methyl-β-Glc seemed to overlap those of Sop_n_s. ESI-MS analysis using phenyl-α-Glc and Sop_2_ detected peaks assigned as the compounds of phenyl-α-Glc linked with one or two Glc units clearly, though the peak corresponding to Glc was small (Fig. S7*B*). This result is consistent with detection of two spots indicated by arrows below the spot of phenyl-α-Glc in the TLC plate (Fig. 2).

### Kinetic analysis of glucosides

We determined the kinetic parameters of the glycosyltransfer activity of IaSGT using Sop_2_ as a donor and various glucosides as acceptors. The enzyme showed remarkably higher activity toward most of the investigated acceptors than that in the absence of the acceptors (Table 2). The *K*_m_ values for the α-glucosides were at the range of 0.044−0.38 mM (approximately 300−2600 times smaller than that of Sop_2_ without the acceptors), and the *k*_cat_/*K*_m_ values for the acceptors were approximately 70−570 times higher than that of Sop_2_ without an acceptor. In the case of β-glucosides, the *K*_m_ values were in the range of 0.021−0.15 mM (approximately 770−5500 times smaller than that of Sop_2_ without the acceptors), and *k*_cat_/*K*_m_ values were approximately 30−800 times higher than that of Sop_2_ without an acceptor. Overall, β-glucosides showed smaller *K*_m_ and *k*_cat_ values than those of α-glucosides. However, the *k*_cat_/*K*_m_ values for acceptors with both types of anomers were within a similar range and were sufficiently large as those of GH enzymes. Therefore, the enzyme can act on a wide range of glucosides with various aryl- and alkyl-groups and both types of anomers as acceptors. In addition, IaSGT comparably acted on amygdalin, a gentiobioside, as an acceptor, though the *K*_m_ and *k*_cat_/*K*_m_ values were rather larger and smaller than those of the β-glucosides, respectively.

**Table 2:** Kinetic parameters of IaSGT for acceptors and donors

Substrate	*k*_cat_ (s^−1^)	*K*_m_ (mM)	*k*_cat_/*K*_m_ (s^−1^ mM^−1^)
Acceptor^a^			
α-Glucosides			
Methyl-α-Glc	1.0 ± 0.1	0.20 ± 0.03	4.9 ± 0.4
Ethyl-α-Glc	0.55 ± 0.02	0.044 ± 0.005	13 ± 1
Phenyl-α-Glc	1.8 ± 0.2	0.22 ± 0.03	8.1 ± 0.6
α-Arbutin	8.7 ± 0.6	0.38 ± 0.05	23 ± 2
pNP-α-Glc	5.0 ± 0.3	0.13 ± 0.02	39 ± 3
Benzyl-α-Glc	0.86 ± 0.05	0.096 ± 0.011	9.0 ± 0.7
2-Naphthyl-α-Glc	3.9 ± 0.3	0.15 ± 0.03	26 ± 3
β-Glucosides			
Methyl-β-Glc	1.9 ± 0.2	0.13 ± 0.03	14 ± 2
n-Octyl-β-Glc	N.D.^b^	N.D.^b^	N.D.^b^
β-Arbutin	1.4 ± 0.1	0.026 ± 0.003	55 ± 5
pNP-β-Glc	0.46 ± 0.02	0.040 ± 0.004	12 ± 1
Gastrodin	0.49 ± 0.02	0.042 ± 0.004	12 ± 1
Salicin	0.88 ± 0.03	0.021 ± 0.003	43 ± 1
Esculin	1.3 ± 0.1	0.045 ± 0.006	29 ± 1
β-Gentiobioside			
Amygdalin	0.35 ± 0.02	0.15 ± 0.02	2.3 ± 0.6
Donor^c^			
Sop_2_	70 ± 3	9.6 ± 1.2	7.3 ± 0.7
Sop_3_	12 ± 1	22 ± 3	0.54 ± 0.04

All experiments were carried out in triplicate. Standard errors are used in the table.

a 0.025 to 0.4 mM acceptors were used. A fixed concentration of Sop_2_ (1 mM) was used as a donor.

b Values were not determined. This might be due to instability caused by n-octyl-β-Glc as a detergent.

c 2.5 to 80 mM donors were used. A fixed concentration of pNP-α-Glc (0.4 mM) was used as an acceptor.

Next, the kinetic parameters for Sop_2_ and Sop_3_ as donors were determined in the presence of pNP-α-Glc as an acceptor in order to investigate donor specificity (Table 2). The *K*_m_ and *k*_cat_ values for Sop_2_ were approximately two times smaller and six times higher, respectively, than those for Sop_3_, resulting in an approximately 14 times higher *k*_cat_/*K*_m_ value for Sop_2_ than that for Sop_3_.

### Overall structure of IaSGT

**Figure 3:**
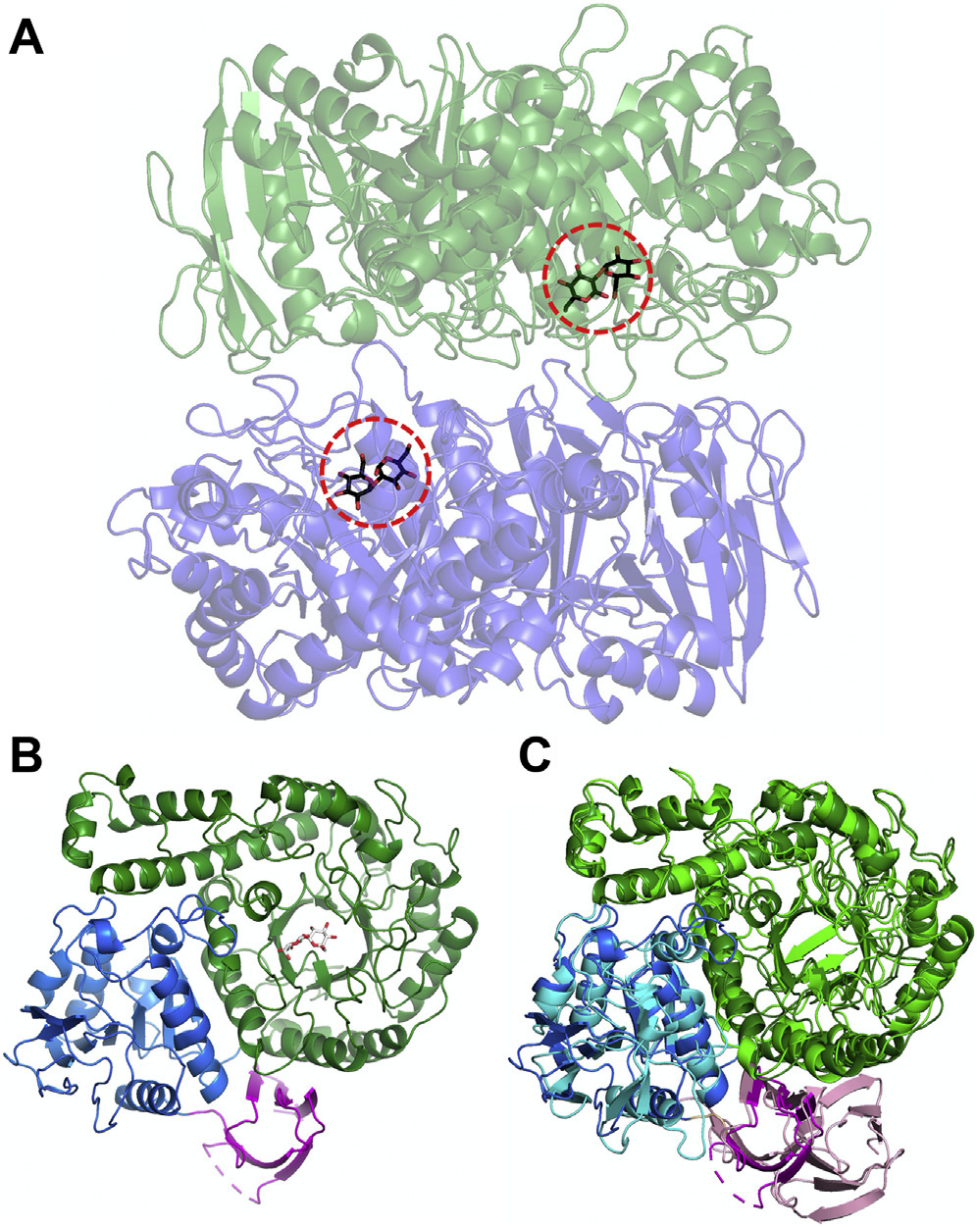
**Overall structure of IaSGT.***A*, the quaternary structure of the WT-Sop_2_ complex is shown as semitransparent *blue* and *dark green cartoons*. The Sop_2_ molecules are shown as *black sticks* and are indicated by *red dashed circles*. *B*, domain constitution of IaSGT. (β/α)_8_ TIM-Barrel domain (residues 6–432), Rossmann fold domain (residues 438–648), and Ig-like domain (residues 653–716) are colored *dark green*, *blue*, and *magenta*, respectively. The Sop_2_ molecule is shown in the TIM-barrel domain as a *white stick*. *C*, superimposition with TkGlmA. The corresponding domains of TkGlmA in IaSGT are shown in *green*, *cyan*, and *light pink*. While the domains of TkGlmA in *green* and *cyan* are classified into the same category according to CATH v4.2 (40), the domain shown in *light pink* is classified into β-galactosidase, domain2 and is apparently larger than that of the corresponding domain in IaSGT. However, the structurally aligned regions in the domains are quite similar in their structures.

First, we determined the ligand-free structure of wild-type IaSGT at 1.75 Å resolution after solving the initial phase by the SAD method (Table S2). Complex structures with various ligands were also solved, as shown in Table S2. The overall conformational change on binding of ligands is small (rmsd, approximately 0.2 Å). Thus, the wild-type (WT)-Sop_2_ complex was used as a representative structure in Figure 3. There are two molecules in an asymmetric unit with almost the same configuration (rsmd between subunits A and B, 0.5 Å) (Fig. 3*A*), which is identical to the biological assembly suggested by the results of size-exclusion chromatography analysis and SDS-PAGE (Fig. S4*B*). Each subunit consists of a (β/α)_8_ TIM-barrel domain (residues 6−432), which is usually found in GH35 enzymes, a Rossmann fold domain (residues 438−648), and an Ig-like domain (residues 653−716) according to CATH v4.2 in RUPEE server (https://ayoubresearch.com/) (41) (Fig. 3*B*). The ligands in the complexes are bound at the center of the (β/α)_8_ TIM-barrel domain.

A structural homology search was performed using the Dali server (http://ekhidna2.biocenter.helsinki.fi/dali/) (42). TkGlmA was found as the enzyme that was most similar to IaSGT (Z-score, 42.6; rmsd, 2.5 Å). IaSGT has a similar quaternary structure and domain configuration to those of TkGlmA, though the C-terminal domain is smaller than that of TkGlmA (Figs. 3*C* and S3). IaSGT has much smaller numbers of hydrogen bonds and salt bridges at the subunit interface (25 and 8, respectively) than those in TkGlmA (58 and 32, respectively), as based on interface analysis with PISA (https://www.ebi.ac.uk/msd-srv/prot_int/pistart.html) (43).

### Binding mode of Sop_2_

**Figure 4:**
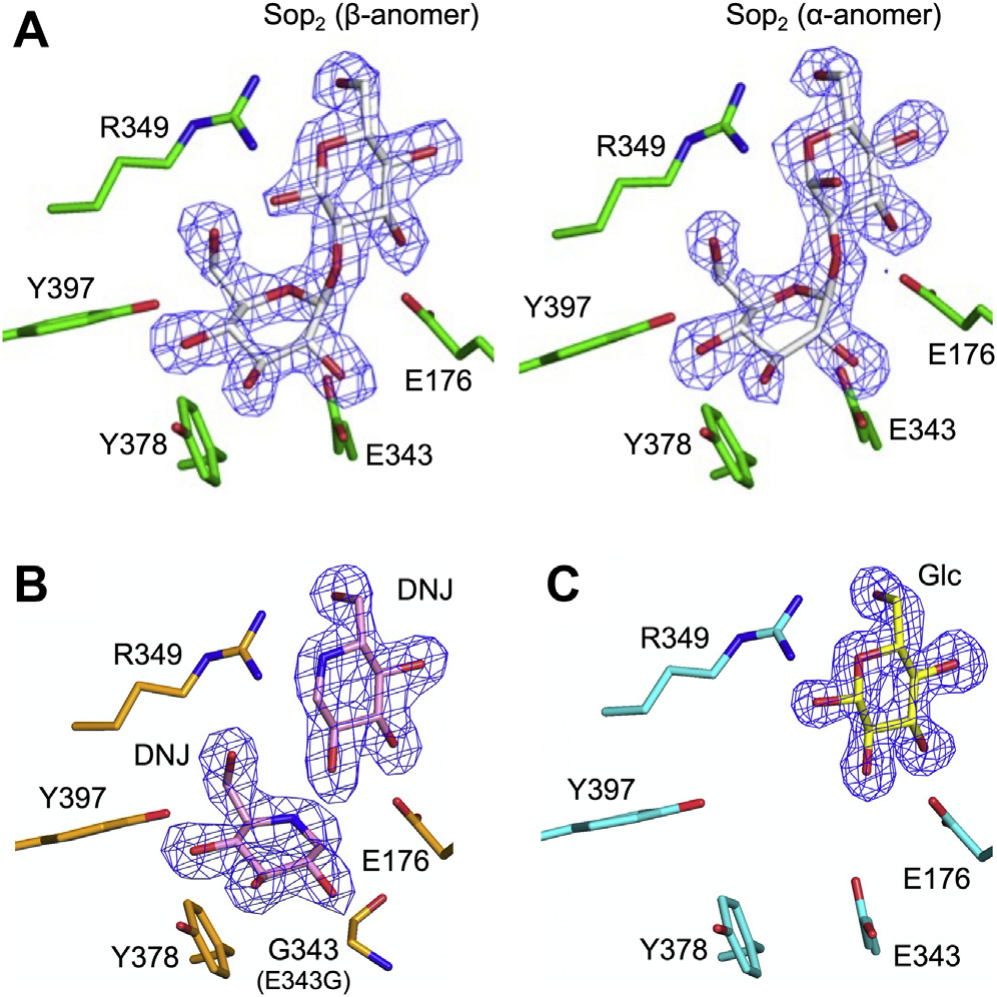
**Electron density of Sop**_**2**_**, DNJ, and Glcin the IaSGT complex structures.** The *F*_o_–*F*_c_ omit maps for the ligands are shown at the 3σ contour level and are shown as *blue meshes*. Main chain atoms are omitted. *A*, WT-Sop_2_ complex (*left* and *right* for subunit A and B, respectively). *B*, E343G-DNJ complex (subunit A). *C*, WT-Glc complex (subunit A). Residues in the three complexes are shown as *green*, *light orange*, and *cyansticks*, respectively, and ligands as *white*, *light pink*, and *yellowsticks*, respectively.

**Figure 5:**
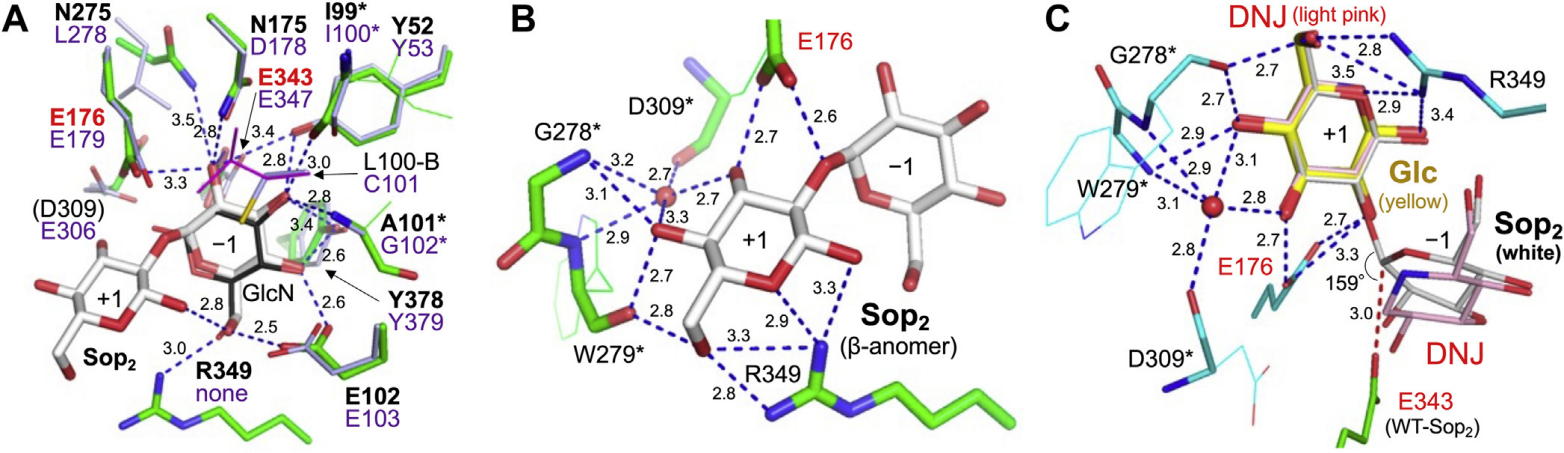
**Binding mode at subsites −1 and +1.** The color usage is the same as in Figure 4. Hydrogen bonds are shown as *blue dashed lines* along with their lengths. Water molecules are shown as *red spheres*. Side chains that do not participate in substrate recognition are shown as *thin sticks*. Main chain atoms are omitted unless needed. Subsite numbers are given near the monosaccharide moieties. *A* and *B*, binding modes of Sop_2_ at subsites −1 (*A*) and +1 (*B*) in subunit A of the WT-Sop_2_ (β-anomer) complex. Residues in *red letters* are candidates for catalysts. *A*, the TkGlmA-GlcN complex (PDB ID, 5GSM) is aligned with the WT-Sop_2_ complex with substrate recognition residues. GlcN in TkGlmA is shown as a *black stick*. Residues in IaSGT and TkGlmA are shown at the *top* and *bottom*, respectively. Residues in *bold letters* in IaSGT indicate that side chains form hydrogen bonds with Sop_2_, while *asterisks* represent that main chain atoms hydrogen-bond with Sop_2_. The residue in parenthesis does not participate in substrate recognition though the corresponding residue in TkGlmA does. Leu100 in subunit A is disordered and the residue in subunit B (L100-B) is shown as a thin *magenta stick*. *C*, superimposition of Sop_2_, Glc, and DNJ. Subunits A of the three complexes are aligned. The *red dashed line* represents the pathway of in-line attack on the anomeric center and its length is also shown.

In order to understand the binding modes of ligands, the complex structures of WT with glucose (Glc) and Sop_2_ and the complex of the E343G mutant with 1-deoxynojirimycin (DNJ) were determined (Fig. 4). The electron densities of Sop_2_ were clearly observed in both subunits of the WT-Sop_2_ complex (Fig. 4*A*). The fitted Sop_2_ molecules in subunits A and B are β- and α-anomers of Sop_2_, respectively. When the GlcN recognition residues in the TkGlmA-GlcN complex are aligned with the corresponding residues in the WT-Sop_2_ complex, the Glc moiety at the nonreducing end of Sop_2_ is well superimposed with the GlcN molecule (Fig. 5*A*), suggesting that Sop_2_ is bound at subsites −1 and +1. Most recognition residues at subsite −1 are conserved in TkGlmA spatially. Cys101 in TkGlmA is replaced by Leu100 in IaSGT, though Leu100 is disordered in subunit A and flips in subunit B. The O6 atom of the Glc moiety is recognized by Arg349, which has no corresponding residue in TkGlmA. One of the 2-hydroxy group recognition residues (Asn175) is replaced by Asp178 in TkGlmA, as described above. This observation implies that these residues are important to distinguish between Glc and GlcN.

One of the most distinctive features of the IaSGT structure is the firm recognition of a Glc moiety at subsite +1. Arg349 hydrogen-bonds with the 6-hydroxy group and O5 atom of the pyranose ring in the Glc moiety (Fig. 5*B*). The β-anomeric hydroxy group is also recognized by the same residue in subunit A, while the corresponding hydroxy (α-anomeric) group in subunit B is not. The 3-, 4-, and 6-hydroxy groups of the Glc moiety form hydrogen bonds with the main chain atoms of Gly278, Trp279, and Asp309, including those mediating a water molecule. Glu176, a candidate for an acid/base catalyst, forms hydrogen bonds with the 2- and 3-hydroxy groups. Therefore, all (or all but anomeric) oxygen atoms in the Glc moiety are recognized by the enzyme, implying narrow specificity to the Glc moiety at subsite +1.

Glu343 and Glu176 are also well superimposed with the nucleophile and acid/base residues of TkGlmA, respectively (Figs. 1 and 5*A*). The activities of E176Q and E343Q were investigated in the presence of 1 mM Sop_2_ as a donor and 0.4 mM pNP-α-Glc as an acceptor. E176Q and E343Q showed less than 0.4% and 0.1% relative activity toward the WT enzyme, respectively, suggesting that Glu176 and Glu343 are an acid/base and a nucleophile, respectively. At subsite −1 in the WT-Sop_2_ complex, the distance between the carboxy group oxygen atom in the Glu343 side chain and the anomeric carbon atom (3.0 Å for both subunits) and the angle formed by the two atoms and the glycosidic bond oxygen atom in the Sop_2_ molecule (159° for subunit A and 165° for subunit B) are suitable for nucleophilic (in-line) attack on the anomeric center of the Glc moiety by Glu343 (Fig. 5*C*). The carboxy group of the Glu176 side chain interacts with the scissile bond oxygen atom in the Sop_2_ molecule (2.6 Å between the two atoms) and is located where *anti*-protonation can occur (44). These results suggest that Glu343 and Glu176 are the nucleophile and acid/base, respectively.

### Binding modes of DNJ and Glc

The electron densities of DNJ molecules were observed only in subunit A in the E343G mutant (Fig. 4*B*). The DNJ molecules are located at subsites −1 and +1 like Sop_2_ in the WT-Sop_2_ complex (Fig. S9). The DNJ molecule at subsite +1 is well superimposed with the corresponding moiety in the WT-Sop_2_ complex. In contrast, the position of the other DNJ molecule shifts to a little below from the corresponding distorted Glc moiety (^3^H_2_ conformation, *φ* = 322.034°, *θ* = 125.390°, Q = 0.532, according to the Cremer–Pople parameter calculator) (45) of the WT-Sop_2_ complex at subsite −1 (Fig. 5*C*). This is probably due to pushing up of the anomeric area by the Glu343 side chain, because the potential distance between the side chain oxygen atom in the carboxy group of Glu343 and the carbon atom corresponding to an anomeric one in the DNJ molecule is too close (1.3 Å). The absence of the side chain of the residue in the E343G mutant enables accommodation of the DNJ molecule without distortion (^4^C_1_ conformation, *φ* = 145.774°, *θ* = 4.556°, Q = 0.579).

When a crystal was soaked in a solution containing Glc, the electron densities of the Glc molecules were clearly observed in both subunits as the β-anomer at subsite +1 at almost the same positions as the DNJ molecule and the Glc moiety of Sop_2_ (Figs. 4*C* and 5*C*). In contrast, Glc is absent at subsite −1 in the complex unlike in the DNJ and Sop_2_ complexes. The potentially too close distance between the DNJ molecule at subsite −1 and the Glu343 side chain suggests that an α-anomeric configuration of a Glc molecule is not allowed at subsite −1. If two Glc molecules bound at both subsites −1 and +1, the 1-hydroxy group (β-anomer) of a Glc molecule at subsite −1 and the 2-hydroxy group of a Glc molecule at subsite +1 would collide. These observations suggest that binding at subsite +1 is obviously stronger than that at subsite −1.

**Figure 6:**
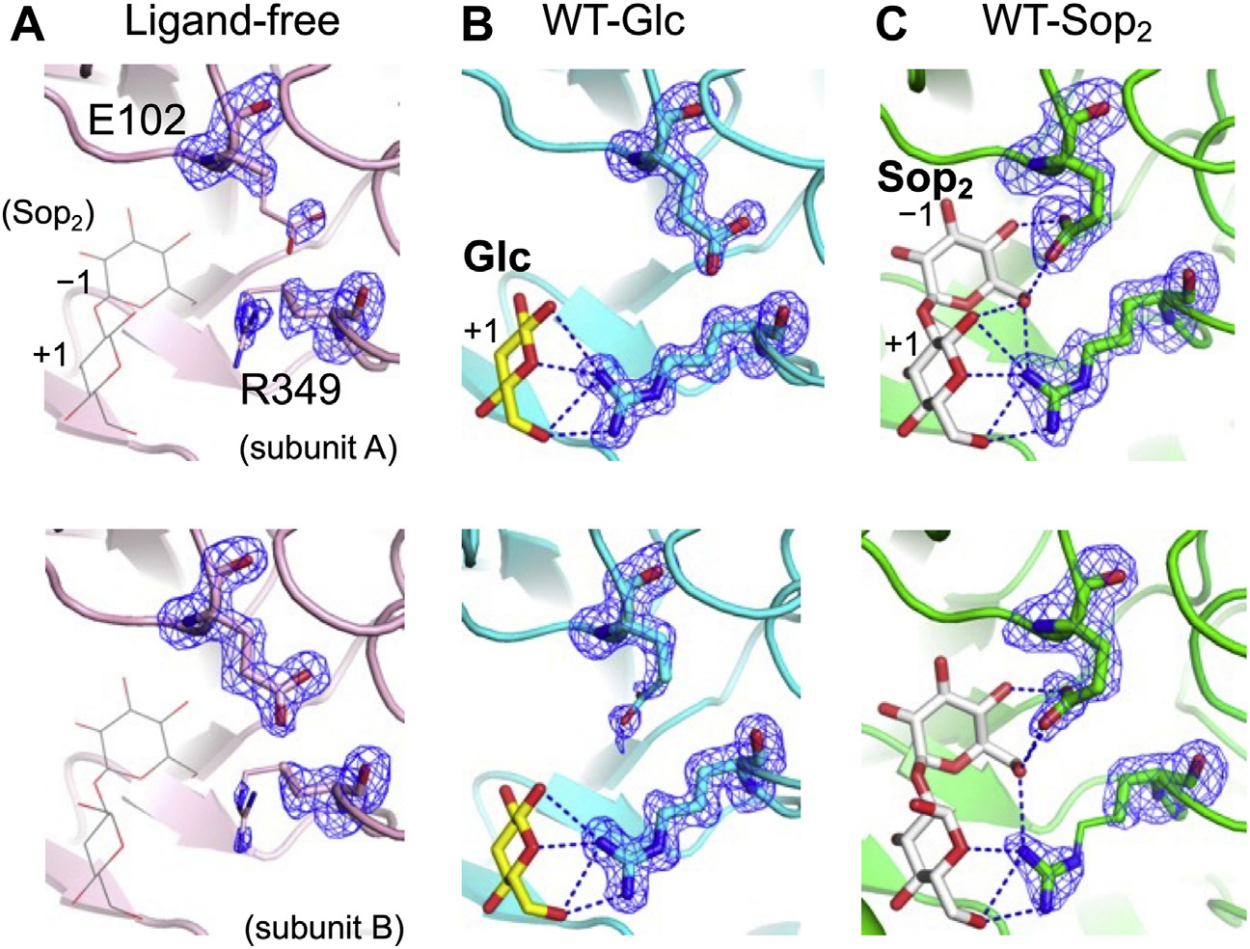
**Comparison of the positions of Glu102 and Arg349 in theligand-free, theWT-Glc, and WT-Sop**_**2**_**structures.** Subunits A and B are shown at the *top* and *bottom*, respectively. The parts where electron densities are poor in the side chains of the residues are shown as *thin sticks*. The numbers around the carbohydrates are the subsite numbers. *A*, the ligand-free structure is shown as a *light pink cartoon* and is superimposed with Sop_2_ shown by a *line* in the WT-Sop_2_ complex. The superimposed ligand is indicated in *parenthesis*. *B* and *C*, the representation of hydrogen bonds and the *F*_o_–*F*_c_ omit maps for Glu102 and Arg349 (*blue meshes*), and color usage for the WT-Glc and the WT-Sop_2_ complexes (a *cyan cartoon* and a *yellow stick*, and a *green cartoon* and a *white stick*, respectively) are the same as in Figures 4 and 5. *C*, the Sop_2_ molecules at the *top* and *bottom* are β- and α-anomers, respectively.

The positions of Glu102 in the complexes with ligands should be noted, since the positions are related to substrate preference, as described later. In the ligand-free structure, the side chain of Arg349 is disordered beyond the C_δ_ atom. The side chain of Glu102 is also disordered (subunit A) or flips out from subsite −1 (subunit B) (Fig. 6*A*). In the WT-Glc complex, Arg349 participates in substrate recognition to give a stable conformation, whereas Glu102 is still disordered or flips out as the ligand-free structure (Fig. 6*B*). Contrarily, in the WT-Sop_2_ complex, the Glu102 residue in each subunit clearly faces subsite −1 to form hydrogen bonds with the Glc moiety, suggesting that the side chain of Glu102 must interact with a Glc moiety at subsite −1 to face in the direction of subsite −1 (Fig. 6*C*).

### Binding mode of Sop_2_ in the E343Q-Sop_2_ complex

**Figure 7:**
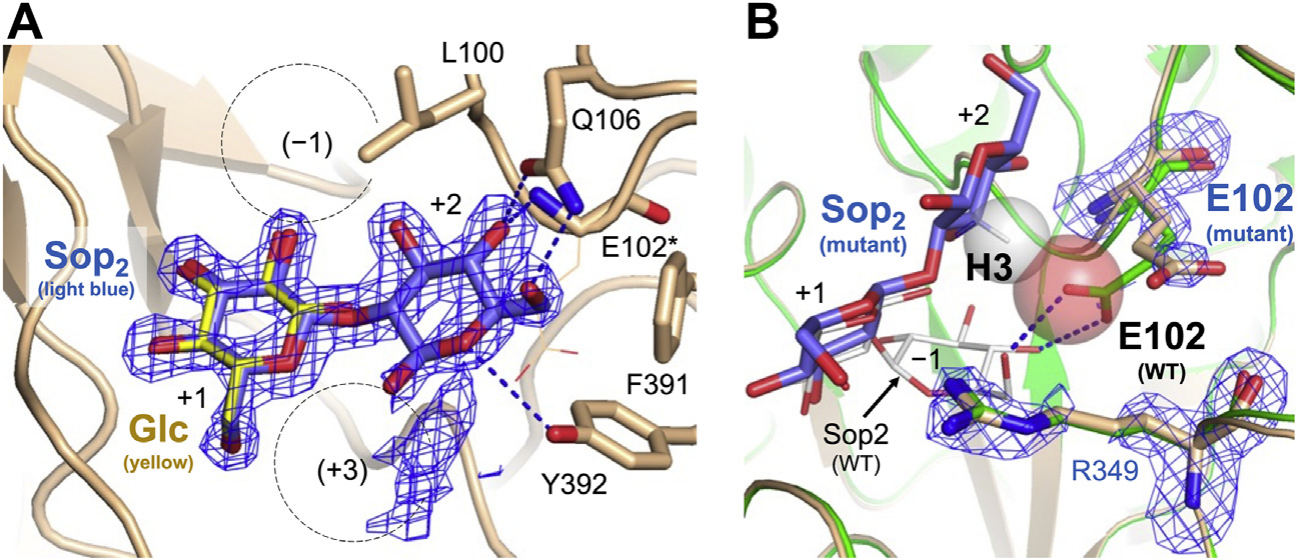
**Binding mode of Sop**_**2**_**in the E343Q-Sop**_**2**_**complex.** The *F*_o_–*F*_c_ omit maps at the 3σ contour level for Sop_2_, Glu102 and Arg349 are shown as *blue meshes*. *A*, electron density of a Sop_2_ molecule. The polypeptide chain is shown as a *light brown cartoon*. Residues involved in recognition of the Glc moiety at subsite +2 are shown as *light brown sticks*. The *asterisk* indicates that only a main chain atom participates in substrate recognition at subsite +2 and the side chain is shown as a *line*. Hydrogen bonds only at subsite +2 are shown as *blue dashed lines*. The length of the hydrogen bond provided by Tyr392 is a little longer than normal ones (3.6 Å). The Sop_2_ molecule is shown as a *light blue stick*. The Glc molecule in the WT-Glc complex (a *yellow stick*) is superimposed in the same way as in Figure 5. Subsite numbers with weak or no electron density are given in *parentheses* and indicated by *dashed circles*. *B*, electron densities of Glu102 and Arg349 in the E343Q-Sop_2_ complex. The WT-Sop_2_ complex is superimposed with the same color usage as in Figure 5 (a *green cartoon* and a *white stick*). Both the E343Q-Sop_2_ and WT-Sop_2_ complexes are shown as *cartoons*. Hydrogen bonds between Glu102 and the Sop_2_ molecule in the WT-Sop_2_ complex are shown in *blue dashed lines*. The potential hydrogen atom covalently bonded with the C3 atom in the Glc moiety at subsite +2 in the E343Q-Sop_2_ complex was generated by PyMOL as a *stick*. The van der Waals radii of the hydrogen atom in the E343Q-Sop_2_ complex and the oxygen atom (the one closer to the hydrogen atom) in the carboxy group of Glu102 in the WT-Sop_2_ complex are shown as semitransparent *white* and *red spheres*, respectively.

In order to understand the binding mode at subsite +2, crystals of WT IaSGT were soaked in a solution containing Sop_3_. However, only the same structure as the Sop_2_ complex was obtained (data not shown), probably due to Sop_2_ production through enzymatic reaction in the crystals. In addition, only poor Sop_2_ complex was obtained when using E343G mutant. Thus, crystals of the E343Q mutant (the mutant of the nucleophile) were soaked in a solution containing Sop_4_ (Fig. 7*A*). Clear electron density fitting a Sop_2_ molecule was observed at subsites +1 and +2 only in subunit A. An electron density beyond subsite +2 is almost absent in the solvent, suggesting that there is no subsite +3 in IaSGT. Importantly, as shown later, electron density fitting ligands was not observed at subsite −1 at all.

The Glc moiety at subsite +2 is hydrogen-bonded with the side chain of Gln106 and the main chain of Glu102. Tyr392 may also participate in binding to the O5 atom in the Glc moiety. The aromatic ring of Phe391 undergoes hydrophobic interaction with the C6 atom in the moiety. The position of the Glu102 side chain should be noted. In the WT-Sop_2_ complex, both the side chain carboxy group oxygen atoms of Glu102 participate in the substrate recognition at subsite −1 (Fig. 7*B*). However, the distance between one of the oxygen atoms in Glu102 and a potential hydrogen atom generated from the C3 atom of the Glc moiety at subsite +2 by PyMOL (2.2 Å) is outer limit for van der Waals distance between nonbonded hydrogen and oxygen atoms. The distance is smaller than the normally allowed one (2.4 Å), as shown as overlapped spheres (46) (Fig. 7*B*). This observation suggests that binding at subsites −1 and +2 competes mildly through Glu102. This competition further suggests that Sop_2_ can be a preferable donor but Sop_n_s (n ≥ 3) that have to bind at subsite +2 cannot, and that Sop_n_s are unfavorable as acceptors, though not completely excluded.

### Binding modes of glucoside acceptors

**Figure 8:**
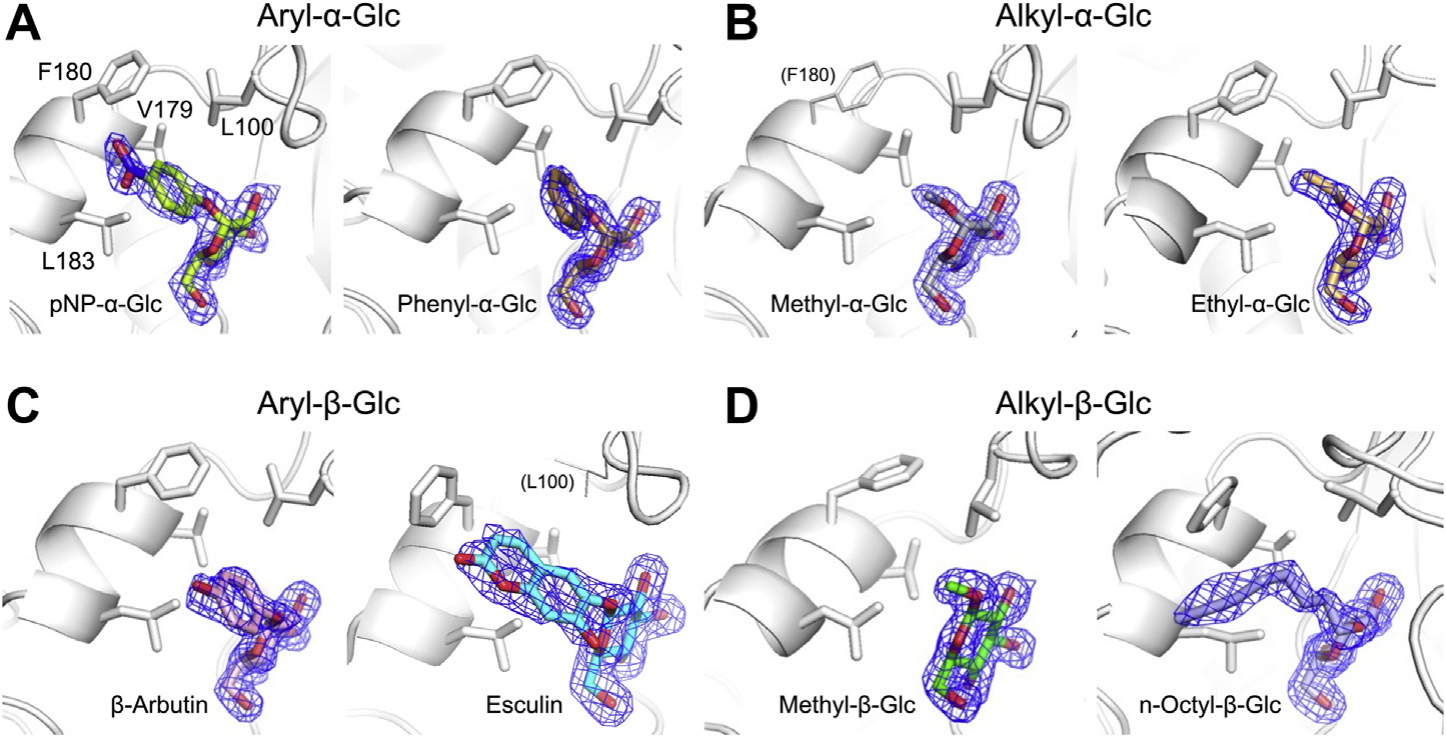
**Electron densities of the glucosides in complex structures with aryl- and alkyl-Glc.** The polypeptide chains are shown as *white cartoons*. Aglycone recognition residues are shown as *white sticks*. The representation of the *F*_o_–*F*_c_ omit maps for the glucosides is the same as in Figure 4 (*blue meshes*). pNP-α-Glc (*A left*), phenyl-α-Glc (*A right*), methyl-α-Glc (*B left*), ethyl-α-Glc (*B right*), β-arbutin (*C left*), esculin (*C right*), methyl-β-Glc (*D left*), and n-octyl-β-Glc (*D right*) are shown in *yellow green*, *dark yellow*, *gray*, *light orange*, *light pink*, *cyan*, *green*, and *light blue*, respectively. Subunits B are shown only for pNP-α-Glc and β-arbutin complexes, while subunits A are shown for the others. Phe180 with weak electron density and Leu100 with disordered electron density in the methyl-α-Glc and esculin complexes, respectively, are shown as *thin sticks*.

**Figure 9:**
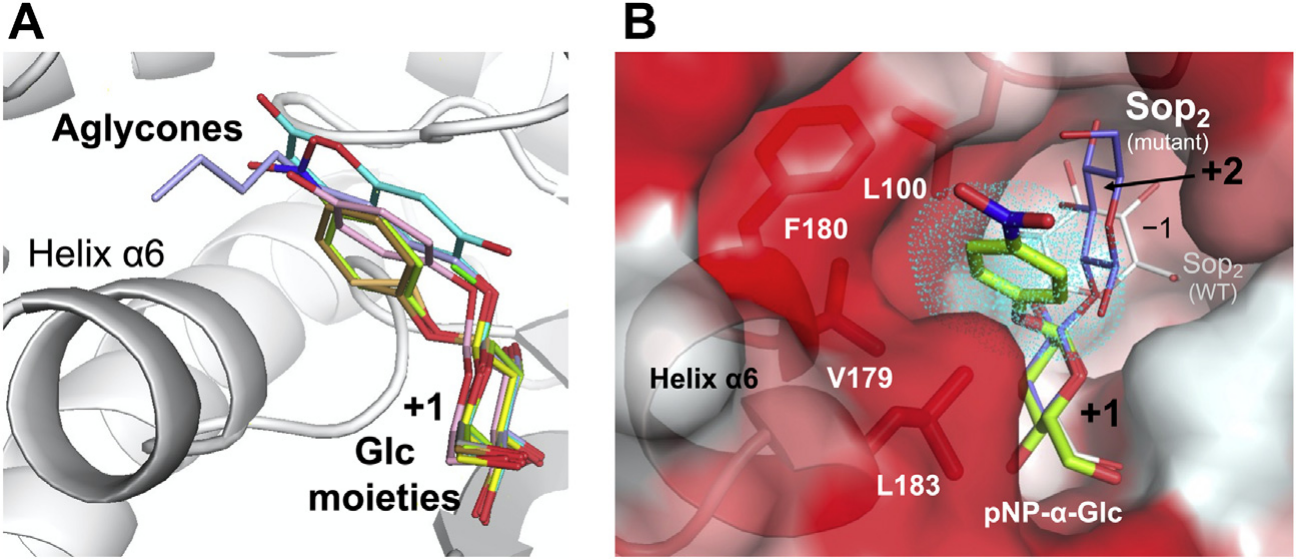
**Binding modes of the hydrophobic pocket.***A*, superimposition of the acceptor glucosides with the Glc molecule in the IaSGT complexes. The color usage is the same as in Figure 8 (a *white cartoon* for IaSGT). *B*, surface representation of the WT-pNP-α-Glc complex. pNP-α-Glc is shown as a *yellow green stick* as in Figure 8*A*. Hydrophobicity is shown as a gradient of *red* to *white* (high to low hydrophobicity) in the surface representation (https://web.expasy.org/protscale/pscale/Hphob.Eisenberg.html) (62). Aglycone recognition residues with hydrophobic residues are colored as in the surface representation and are shown as *sticks*. The polypeptide chain around these residues is shown as a *cartoon* with the same color usage as for the surface representation. The Sop_2_ molecules in the WT-Sop_2_ and the E343Q-Sop_2_ complexes are superimposed and are shown as thin *white* and *light blue sticks*, respectively, with the same color usage as in Figure 7. The van der Waals radii of aromatic carbon atoms are shown as *cyan dots*.

We determined the complex structures of WT IaSGT with various glucoside acceptors listed in Table S2 to elucidate the binding modes of the acceptors. Aryl- and alkyl-glucosides (both anomers) with well-observed electron densities at their aglycones are shown in Figure 8. The Glc moieties of all these acceptors tightly bind to subsite +1 at almost the same position and orientation as in the WT-Glc complex (Fig. 9*A*). Though the positions of the aglycones deviate from each other, all aglycones shown in the figure bind within the same specific area.

In subunit B of the pNP-α-Glc complex, Val179, Phe180, and Leu183 in helix α6 and Leu100 form a hydrophobic dent to interact with the aromatic ring in pNP-α-Glc (Fig. 9*B*). Such hydrophobic interactions are also found in complexes with various aryl- and alkyl-glucosides regardless of the anomer. This hydrophobic hollow is clearly different from subsite +2 in position and is likely to be too small for monosaccharide moieties to be accommodated, judging from the van der Waals spheres of aromatic ring atoms fitting the hollow (Fig. 9*B*).

**Figure 10:**
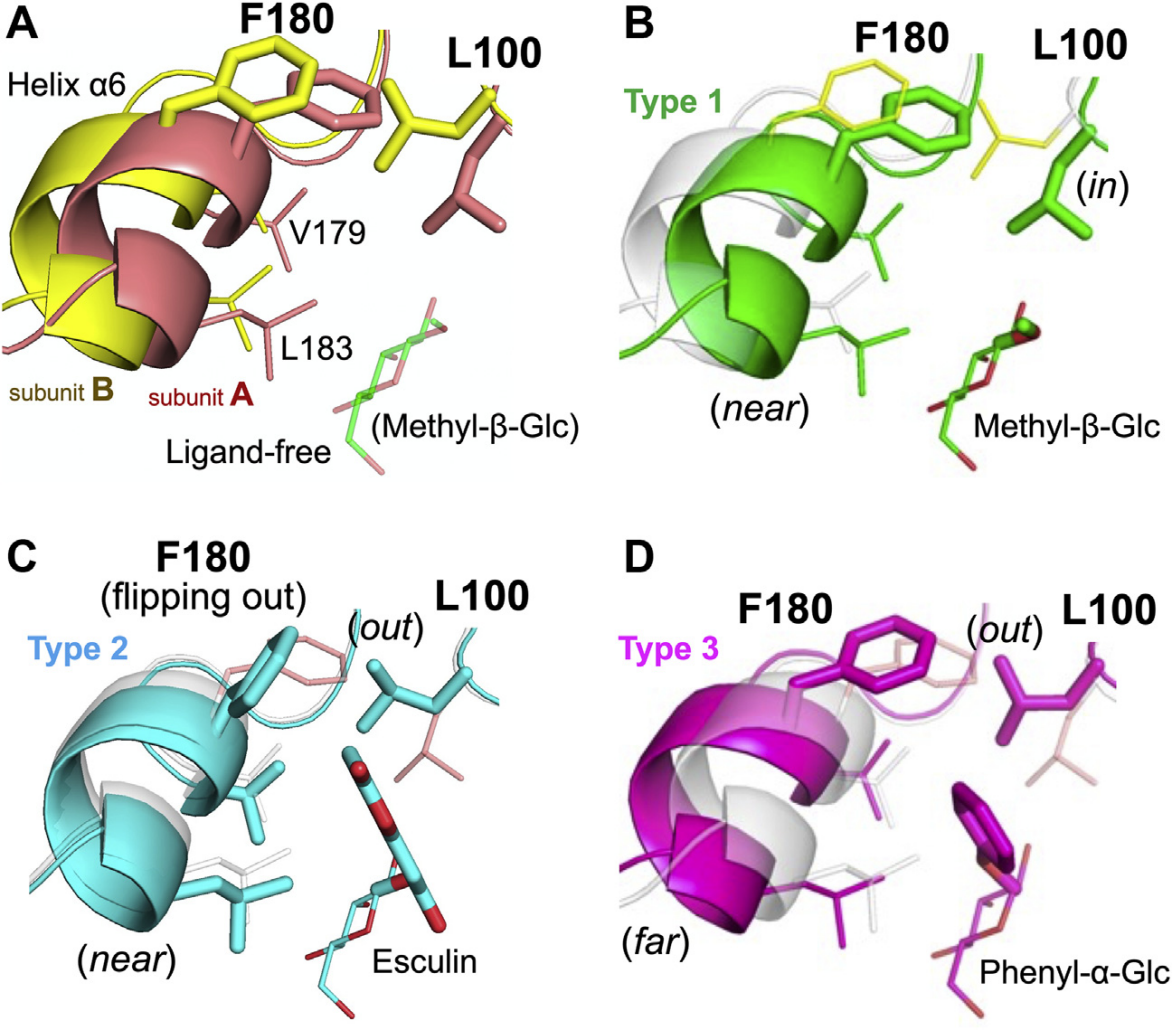
**Three conformational patterns of the hydrophobic pocket**. *A*, subunits A and B of the ligand-free structure are shown in *light brown* and *yellow*, respectively. Residues constituting the hydrophobic pocket are shown as *sticks*. Leu100 and Phe180 are *thick*, and the others are *thin*. Methyl-β-Glc is superimposed as a semitransparent *thin green stick*. *B*–*D*, polypeptide chains and glucosides are shown in the same colors as the corresponding glucosides in Figure 8 (*green*, *cyan*, and *magenta* for *B*–*D*, respectively). Leu100, Phe180 and aglycone moieties are shown as *thick sticks*. Val179, Leu183, and Glc moieties are shown as *thin sticks*. *B*, type 1 conformation. Subunit B of the ligand-free structure is superimposed semitransparently. Leu100 and Phe180, and helix α6 in the subunit are shown as *thin sticks* and a *white cartoon*, respectively. *C* and *D*, type 2 (C) and type 3 (*D*) conformation. Subunits A is shown translucently as in the same way as subunit B in (*B*).

One of the noticeable features of these complex structures is the side chain of Leu100 and helix α6. There are two rotamers for the side chain of Leu100; one faces subsite +1 (*in*), and the other faces in the opposite direction (*out*). There are also two possible positions for helix α6; one is close to the substrate pocket (*near*), and the other is a little far from the pocket (*far*). Three types of combinations are found in the complex structures, as summarized in Table 3. Subunit A of the ligand-free structure adopts a conformation in which both the Leu100 side chain and helix α6 are close to the substrate pocket (type 1), while subunit B adopts a conformation in which both are away from the substrate pocket (type 3) (Fig. 10*A*). Subunit A of methyl-β-Glc, Sop_2_ and DNJ complexes, and both subunits of the Glc complex adopt the type 1 conformation, which is likely to be observed if the aglycone moiety of ligands is absent or small (Fig. 10*B* and Table 3). Large aglycone moieties are likely to push the Leu100 side chain away from subsite +1. The side chains of Leu100 (*out*) and Phe180 in helix α6 (*near*) are potentially located within the range of steric hindrance (around 1.5 Å). To avoid such hindrance, both side chains are disordered (*e.g.*, subunit A of the pNP-α-Glc complex) or the side chain of Phe180 flips out (*e.g.*, subunit A of the esculin complex) for type 2 (Fig. 10*C*). Otherwise, helix α6 is pushed out by Leu100 for type 3 (*e.g.*, both subunits of phenyl-α-Glc) (Fig. 10*D*). The dent near helix α6 does not accommodate carbohydrate moieties due to its small size, but its conformational variety may allow various aglycones.

**Table 3:** Patterns of conformations at the substrate pocket

Ligand^a^	Subunit	Conformations of Leu100 and helix α6				
				Type 1	Type 2	Type 3
		Leu100		in	out	out
			Helix α6 (Phe180)^b^	near	near	far
Ligand-free	A			○		
	B					○
Glc	A			○		
	B			○		
Sop_2_	A	Disorder	(Disorder)			○
	B			○		
DNJ^c^	A			○		
<α-Glucosides>						
Methyl-α-Glc	A		(weak)			○
Ethyl-α-Glc	A					○
Phenyl-α-Glc	A					○
	B					○
pNP-α-Glc	A	Disorder	(Disorder)		○	
	B					○
<β-Glucosides>						
Methyl-β-Glc	A			○		
	B	Disorder				○
n-Octyl-β-Glc	A		(Flip out)		○	
	B					○
β-Arbutin	B					○
Esculin	A	Disorder	(Flip out)		○	

a In the case of both subunits of α-arbutin, benzyl-α-Glc, 2-naphthyl-α-Glc, gastrodin, pNP-β-Glc, salicin, and amygdalin, and for subunits that are not shown in the table, the electron densities of the aglycone moieties of the ligands were partially obscure, the ligands were not observed, or binding modes of the ligands were apparently artificial due to potential steric collision with the Glu102 side chain participating in binding at subsite −1 (data not shown).

b The electron density states of the Phe180 side chain are shown in parentheses.

c The E343G mutant was used for the complex with DNJ. The WT enzyme was used for the other structures.

## Discussion

### Classification of IaSGT

**Figure 11:**
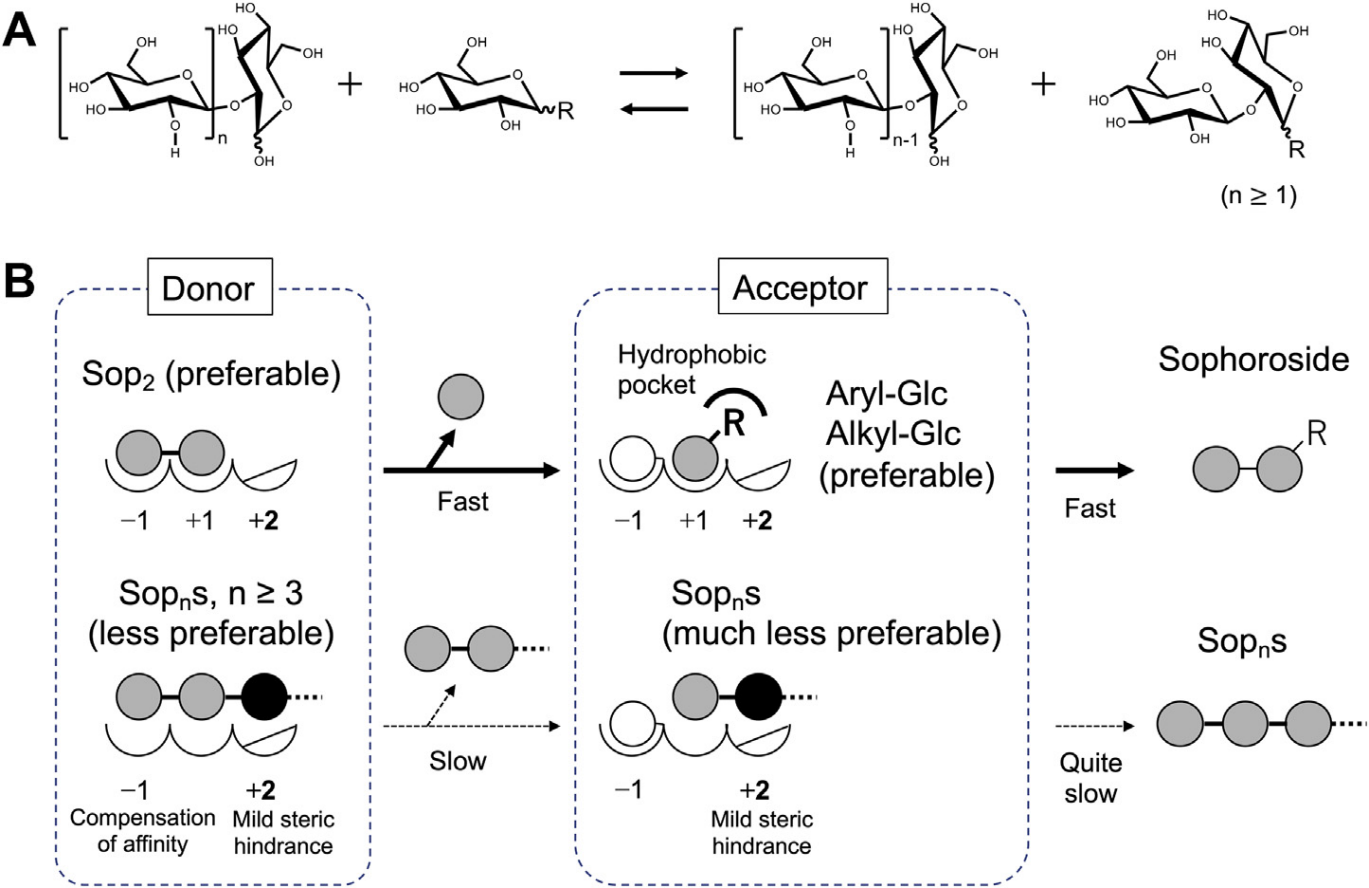
**Schematic representation of the reactionand the mechanism of substrate preferenceof IaSGT.***A*, IaSGT transfers a glucose moiety from the nonreducing end of Sop_n_ to a glucoside and links it through a β-1,2-glucosidic bond. *B*, Glc molecules and moieties are shown as *gray circles*. The colors of ones bound at subsite +2 and covalently bound with the enzyme are *black* and *white*, respectively. Subsites in the enzyme are shown as *semicircles* and the numbers are assigned for the subsites. Unfavorable factors for binding are indicated by *slash lines* in subsite +2. The hydrophobic pocket is shown as a *bold partial circle*. Letter R represents an aryl or alkyl group.

In this study, we found that IaSGT was a glycosyltransferase acting on the β-1,2-glucosidic bonds. The enzymatic reaction and structure–function relationship are shown schematically in Figure 11. Interestingly, suitable acceptors were not Sop_n_s but various aryl- and alkyl-glucosides, though gentiobiosides are likely to be allowed as acceptors as well based on the results of assaying for amygdalin. Many of the investigated glucosides are natural compounds, for example, ethyl-α-Glc is one of the umami compounds found in fermented foods such as liquor and some seasonings; β-arbutin, gastrodin, salicin, and esculin are found in plants (47, 48, 49, 50). Such wide acceptor specificity among glucosides is attributed to the strong recognition of the Glc moiety at subsite +1 (Figs. 5 and 8) and the mobile hydrophobic region accommodating aglycones (Fig. 9*B*). The side chain of Glu102 binding to the Glc moiety at subsites −1 makes Sop_n_s unfavorable as acceptors due to steric hindrance as to subsite +2, though the hindrance is not too strict to inhibit production of Sop_n_s completely (Figs. 7*B* and 11*B*). This hindrance also makes IaSGT preferable for Sop_2_ as a donor than the other Sop_n_s (Fig. 11*B*; Tables 1 and 2). However, Sop_3_ cannot be excluded sufficiently as a donor from an enzymological point of view unlike in the case of Sop_n_s as acceptors (Tables 1 and 2). In the case of donors, binding at subsite −1 compensates for the disadvantage at subsite +2 for binding. This perspective suggests that it is more appropriate to regard Sop_n_s rather than only Sop_2_ as donors for classification of enzymatic reactions.

The reaction mode of IaSGT comprises transfer of a β-1,2-linked glucose unit without any hydrolytic activity, unlike some GH35 hydrolases possessing glycosyltransfer activity such as β-galactosidase from *Aspergillus niger* (51). Though IaSGT is similar to SOGP in the transfer of a β-1,2-linked glucose unit, there is no space necessary for accommodation of an inorganic phosphate below the anomeric center attacked by a nucleophile. While IaSGT prefers aryl- and alkyl-glucoside regardless of the anomer as acceptors, SOGP requires sophorose as a minimum acceptor in the synthetic reaction. In addition, IaSGT is presumed to exhibit an anomer-retaining mechanism based on the conservation of catalytic residues in the GH35 family, while SOGP exhibits an anomer-inverting mechanism. Overall, IaSGT is a novel enzyme that should be given a new EC number. We propose β-1,2-glucooligosaccharide:d-glucoside β-d-glucosyltransferase as a systematic name and β-1,2-glucosyltransferase as an accepted name (Fig. 11*A*).

### Comparison of substrate recognition residues with those of GH35 enzymes

Characterization and structural analyses of IaSGT revealed residues important for substrate recognition and catalysis. The recognition residues at subsite −1 and catalytic residues (Y52, E102, N175, E176, N275, E343, and Y378) in IaSGT are well conserved among IaSGT homologs, implying that IaSGT homologs share the same specificity at subsite −1 and a fundamental reaction mechanism.

One of the interesting issues regarding GH35 is how the enzymes distinguish Glc/GlcN and Gal (O4 epimers). Rotamers of 6-hydroxy groups should also be taken into account, since the rotamer preference of the 6-hydroxy groups is affected by the orientations of 4-hydroxy groups to avoid proximity of the O4 and O6 atoms. Preferable conformations of 6-hydroxy groups of Glc and Gal are *gg* and *tg*, respectively. The O4 and O6 atoms are recognized by Glu102 and Tyr378 in IaSGT. The enzyme has Tyr397, which can bind to the O6 atom in the *tg* rotamer potentially. Though Tyr397 is not conserved among IaSGT homologs (Fig. S10), this residue is conserved in the β-galactosidase from *Streptococcus pneumoniae* TIGR4 (SpBgaC) (Fig. S11 right and left), implying the need for other factors to differentiate the specificity at subsite −1. Leu100, a residue corresponding to Cys96 in the β-galactosidase spatially, may avoid the O4 axial orientation due to its hydrophobicity. Arg349 recognizing O6 with a *gg* rotamer cannot form a hydrogen bond with O6 with the *tg* rotamer potentially, which may also affect the specificity for O4 epimers. In the case of TkGlmA, Cys101 is located at the position corresponding to Leu100 in IaSGT as well as SpBgaC (Fig. S11 middle), implying that Cys101 is unlikely to be involved in the distinction of O4 epimers (39). Instead, Trp308 at the bottom of the pyranose ring is likely to make TkGlmA unpreferable for the *tg* rotamer of a 6-hydroxy group. In addition, TkGlmA does not possess a residue corresponding to Tyr305 in SpBgaC recognizing an O6 atom with the *tg* conformation.

### Diversity of IaSGT homologs

It should be noted that there is interesting diversity at subsite +1 and the aglycone-binding region among IaSGT homologs. The subsite firmly recognizes a Glc moiety by many residues and shows the strongest affinity for the Glc moiety in IaSGT based on structural observation (Figs. 4*C* and 5*C*). Among the recognition residues, Arg349 is the key for substrate specificity. Arg349 provides its side chain for substrate binding, whereas the other bindings are provided by main chain atoms (Gly278, Trp279, and Asp309) or by a catalyst that cannot be replaced by the other residues (Glu176) (Fig. 5). Nevertheless, Arg349 is not conserved among many IaSGT homologs (Fig. S10).

IaSGT also has a hydrophobic region for the binding of aglycones. The residues (Leu100, Val179, Phe180, and Leu183) recognizing alkyl and aryl groups in the acceptors are conserved as hydrophobic ones among the homologs. Among them, Leu100 plays a unique role in the conformational change of the aglycone recognition region (Fig. 9). However, Leu100 is replaced by a Met residue in many of the homologs (Fig. S10). Such differences among IaSGT homologs make us expect diversity in substrate specificity or preference.

### Speculated physiological role of the IaSGT gene cluster

The *IaSGT gene* forms a gene cluster with the genes encoding carbohydrate transporters and GH family members (Fig. S1). The speculated physiological roles of these proteins are shown in Fig. S12. Extracellular β-1,2-glucans may be hydrolyzed by GH144 IALB_1179 protein to Sop_n_s or shorter β-1,2-glucans, since this protein was predicted to be located at the extracellular or cell inner membrane. The products might be transported into the periplasm by a TonB-dependent transporter, though there is no biochemical evidence of close homologs. Sop_n_s can be further degraded by GH144 homologs and be transported into the cytoplasm by an ABC transporter. IALB_1187 is a homolog of the Sop_n_s-binding protein from *L. innocua* (25). IALB_1177 is a homolog of a C-terminal SOGP domain in a cyclic β-1,2-glucan synthase, the protein being expected to release Sop_2_ as a final product. However, there is no β-glucosidase homolog in the gene cluster, implying that Sop_2_ is supplied for IaSGT as a donor.

## Conclusion remarks

We found that IaSGT is an enzyme exhibiting a novel glycosyltransfer reaction. It is intriguing that various aryl- and alkyl-glucosides with both anomers were acceptors for the enzyme. Furthermore, X-ray crystallographic analysis revealed structural features for substrate specificity. This report is also the first β-glucoside-acting enzyme and the first glycosyltransferase in the GH35 family. Glycosyltransfer reactions are of use for oligosaccharide synthesis, but glycosyltransferases in GH families share their reaction mechanisms with glycoside hydrolases basically. Complete comprehension of their reaction mechanism is required to control conversion between transferases and hydrolases freely, though this is still an open issue. Our findings are important biochemical data for understanding the diversity of CAZymes and a fundamental structural basis for further investigation of the profound enigma of the reaction mechanisms of CAZymes.

## Experimental procedures

### Phylogenetic analysis

The amino acid sequences of the characterized GH35 enzymes were retrieved from the CAZy database. The homologous sequences of IaSGT and GlmAs were obtained by means of Protein BLAST (https://blast.ncbi.nlm.nih.gov/Blast.cgi). The sequences were aligned using MUSCLE, and the phylogenetic tree was constructed and visualized by the maximum likelihood method using MEGA X version 10.2.5 (52).

### Cloning, expression, and purification

The gene encoding IALB_1185 protein was amplified by the PCR method using KOD -plus- (TOYOBO) and the genomic DNA of *I. album* (DSM19864) purchased from DSMZ as a template with the primers listed in Table S3. The amplified gene was inserted into the NdeI and XhoI sites of the pET30a(+) vector (Novagen). Mutants of IaSGT were generated using a PrimeSTAR Mutagenesis Basal Kit (TakaraBio) with the primers listed in Table S3. The constructed plasmid was transformed into *E. coli* BL21(DE3). The transformant was cultivated in LB medium containing 30 μg/ml kanamycin at 37 °C until the cell culture reached the log phase (A_600_ ∼0.7), followed by induction using 100 μM IPTG at 20 °C with shaking at 200 rpm overnight. For expression of selenomethionine-labeled IaSGT, the plasmid was introduced into *E. coli* B834(DE3), and the transformant was cultivated in LeMaster medium containing 30 μ g/ml kanamycin. The recombinant protein expressed as a his_6_-tag fusion protein was extracted from *E. coli* cells by sonication and purified by affinity chromatography using a HisTrap FF crude column (GE Healthcare, US) (linear gradient of 0–500 mM imidazole), followed by a HiTrap Butyl HP column (GE Healthcare) (linear gradient of 1.5–0 M ammonium sulfate). The buffer used for purification was 50 mM MOPS (pH 7.5) containing 100 mM NaCl. The recombinant protein was purified to homogeneity by SDS-PAGE. The IALB_1185 solution was dialyzed against 5 mM MOPS buffer (pH 7.0) containing 100 mM ammonium sulfate and 5 mM DTT. The salt and reductant in the buffer were used to prevent aggregation of the protein. The concentration of the purified IaSGT solution was calculated from the absorbance at 280 nm using theoretical molecular mass and extinction coefficient of the recombinant IaSGT (84,709.883 and 113,080 M^−1^ cm^−1^, respectively) (53).

### Carbohydrates used as substrates

Cel_2-5_ and Lam_2-5_ were purchased from Megazyme. Sucrose, maltose, l-arabinose, d-fructose, d-galactose, d-xylose, d-mannose, l-rhamnose, and d-gluconate were purchased from Nacalai Tesque. Sop_2-5_ were prepared using SOGP from *L. innocua* and SGL from *Chitinophaga pinensis* (23, 28, 29). Lactose, α,α-trehalose, melibiose, esculin, d-amygdalin, methyl-α-Glc, ethyl-α-Glc, benzyl-α-Glc, pNP-α-Glc, and pNP-β-Glc were purchased from FUJIFILM Wako Chemical Corporation. Gentiobiose, d-talose, and gastrodin were purchased from Tokyo Chemical Industry. d-Gulose and 2-naphthyl-α-Glc were purchased from Toronto Research Chemicals. α-Arbutin was purchased from Ezaki Glico. n-Octyl-β-Glc and methyl-β-Glc were purchased from DOJINDO LABORATORIES and Merck, respectively. Salicin and β-arbutin were purchased from Combi Blocks.

### Analysis of the effects of pH and temperature

The effect of pH on the enzymatic activity of IALB_1185 was determined by measuring glycosyltransfer activity toward Sop_3_ in various pH buffers (Briton-Robinson buffer, pH 3–12, and sodium acetate buffer, pH 4.5–5.5). Each reaction was performed in a reaction mixture (50 μl) containing 20 mM Sop_3_ and 5.6 μg of IaSGT at 37 °C for 60 min. Similarly, the effect of temperature was determined by measuring the glycosyltransfer activity toward Sop_3_ at various temperatures (20–80 °C). Each reaction was performed in the reaction mixture containing 20 mM Sop_3_ and 5.6 μg IaSGT in 20 mM sodium acetate buffer (pH 5.0) for 60 min. The enzymatic activity was determined by measuring the concentration of Sop_2_ released from Sop_3_ by means of glycosyltransfer activity. Sop_2_ in the sample solution was hydrolyzed to glucose with 0.1 mg/ml HjCel3A (54), which can hydrolyze only Sop_2_ among Sop_n_s, in 100 mM sodium acetate buffer (pH 5.5) at 40 °C for 30 min. The amount of glucose in the mixture was determined by the GOPOD method (24), and the concentration of Sop_2_ derived through glycosyltransfer activity was calculated. All experiments were carried out in triplicate.

### Size-exclusion chromatography

IaSGT (0.5 mg, 500 μl) was loaded onto a Superdex 200 10/300 Gl column (GE Healthcare) equilibrated with 5 mM MOPS buffer (pH 7.5) containing 100 mM ammonium sulfate and 1 mM DTT. Ovalbumin (44 kDa), conalbumin (75 kDa), aldolase (158 kDa), ferritin (440 kDa), and thyroglobulin (669 kDa) in a Gel filtration Calibration kit (GE Healthcare) were used as standard proteins. Blue dextran 2000 (2000 kDa) was used to determine the void volume of the column.

### Substrate specificity of IaSGT

We examined the activity of recombinant IaSGT toward various sugars (Cel_2−5_, Lam_2−5_, Sop_2−5_). The reaction mixtures, comprising 10 mM carbohydrate, 70 μg/ml IaSGT (0.2 mg/ml for maltose, gentiobiose, melibiose, sucrose, and lactose) in 50 mM sodium acetate (pH 5.0), were incubated at 30 °C overnight. Each sample solution or marker containing 0.5% each carbohydrate (0.5 μl) was spotted onto a TLC plate. The TLC plates were developed with 75% (v/v) acetonitrile in water. After soaking in 5% (v/v) sulfuric acid in methanol, the TLC plates were heated until bands were visualized sufficiently. The reaction for Figure 1*E* was performed using 10 mM Sop_5_ and 0.1 mg/ml IaSGT. A marker was prepared by the reaction using SOGP from *Enterococcus italicus* (approximately 0.3 mg/ml) (55) in the presence of 1% Sop_n_s mixture (hydrolysates of β-1,2-glucan by SGL from *C. pinensis*) and approximately 10 mM sodium phosphate (pH 7.0) at 37 °C overnight. Each sample solution (1 μl) or the marker (0.5 μl) was spotted on the TLC plate, and the plate was developed twice.

### TLC analysis for investigation of acceptors

For exploration of acceptor substrates of IaSGT, we used the IaSGT E343G mutant as a glycosynthase and the WT enzyme. The reaction mixture comprising 90 μg/ml IaSGT E343G, 10 mM α-GlcF, and 10 mM various glucosides in 20 mM sodium acetate buffer (pH 5.0) was incubated at 30 °C overnight. Similarly, each reaction mixture comprising 90 μg/ml IaSGT (WT), 10 mM Sop_2_, and 10 mM various glucosides in 20 mM sodium acetate buffer (pH 5.0) was incubated at 30 °C overnight. Reaction products were visualized by TLC analysis as described above.

### NMR analysis

The enzymatic reaction was performed in a reaction mixture comprising 350 μg/ml IaSGT, 50 mM Sop_3_, and 5 mM sodium acetate buffer (pH 6.0) overnight. The reaction product was purified by size-exclusion chromatography using a Toyopearl HW-40F column (approximately 2 L gel), as described previously (23). Briefly, after the injection of the reaction mixture (approximately 10 ml), the sample was eluted with distilled water. The eluates were fractionated into 10 ml-portions, and the fraction containing only Sop_4_ was lyophilized. The resultant powder was dissolved in D_2_O, and acetone was added as a standard for calibration of chemical shifts. The chemical shifts were recorded relative to the signal of the methyl group of the internal standard acetone (2.22 ppm). As a reference, Sop_4_ was also dissolved in the same solvent. ^1^H-NMR spectra were recorded using a Bruker Advance 400 spectrometer (Bruker BioSpin).

### ESI-MS analysis

The enzymatic reactions (100 μl) were performed overnight based on the description on the reaction for TLC analysis except that 5 mM sodium acetate (pH 5.0) was used as a buffer. Amberlite MB4 (Organo) was added to each sample to remove ionic compounds. After the solution was collected, 60 μl of water was added to the beads, and the wash solution was also pooled. The samples were diluted 100 times with the solution (methanol/water = 1/1, v/v) containing 5 mM ammonium acetate. After filtration, the samples were loaded on the Sciex X500 R QTOF (Sciex) in positive mode at the flow rate of 20 μl/min.

### Assay of glucosyltransferase activity

Reaction mixtures comprising 82.6 μg/ml IaSGT and various concentrations of Sop_n_s (0.5–40 mM Sop_2_, 0.5–40 mM Sop_3_, 0.5–40 mM Sop_4_, or 0.5–40 mM Sop_5_) in 20 mM acetate-Na buffer (pH5.0) were incubated at 37 °C for 1 h, and then the reaction was stopped by heat treatment at 100 °C for 5 min. To determine the kinetic parameters of IaSGT for Sop_2–5_, Glc concentrations in the samples were determined by the GOPOD method based on the manufacturer’s instructions (Megazyme) after the treatments described below. Glc was used as a standard. To determine activity toward Sop_2_, the concentration of glucose released from Sop_2_ was measured. For activity toward Sop_3_, Sop_2_ released from Sop_3_ was hydrolyzed to Glc with HjCel3A. To determine the kinetics for Sop_4_, Sop_5_ generated by IaSGT was hydrolyzed to Sop_2_ and Sop_3_ with SGL from *C. pinensis* (0.12 mg/ml). Then the released Sop_2_ was treated as in the assay for Sop_3_. To determine activity toward Sop_5_, the reaction products were reduced using a one-fifth volume of 1 M NaBH_4_. The same volume of 1 M acetate as that of the NaBH_4_ solution was added to each sample to neutralize NaBH_4_. Then, the samples were treated with 0.12 mg/ml of SGL from *C. pinensis* at 40 °C for 20 min to release Sop_2_ from the reduced Sop_6_. The released Sop_2_ was quantified in the same way as for the assay for Sop_3_. When the effect of an acceptor on activity toward Sop_2_ was examined, the assay was performed using 35 μg/ml IaSGT in the presence of 2 mM Sop_2_ and 1 mM acceptor. The accepters used were mannose, d-gulose, Gal, d-xylose, Cel_2_, Lam_2_, gentiobiose, sucrose, maltose, α,α-trehalose, lactose, l-arabinose, l-rhamnose, fructose, and gluconate.

To determine the kinetic parameters for glycosides as acceptors, colorimetric determination and enzymatic reactions were carried out as described below. The reaction mixture comprised appropriate concentrations of IaSGT, various concentrations of glycosides, 100 U/ml hexokinase, 100 U/ml G6PDH, 1 mM ATP, 1 mM thio-NAD^+^, and 10 mM MgCl_2_ in 20 mM sodium acetate buffer, pH 5.0. Each reaction mixture was incubated at 37 °C, and the increase in absorbance at 398 nm derived from thio-NADH was monitored for 10 min. In the reaction with Sop_3_ as an acceptor, HjCel3A (0.1 mg/ml) was added to the reaction mixture to hydrolyze Sop_2_ to glucose. The extinction coefficient of the assay was determined to be 11,900 M^−1^ cm^−1^ according to the manufacturer's instructions (https://www.oyc.co.jp/bio/IVD_research/coenzyme/ThioNAD.html). Assaying of the E176Q and E343Q mutants was carried out in the same way as for the coupling method described above in the presence of 1 mM Sop_2_ and 0.4 mM pNP-α-Glc. All kinetic parameters in the study were determined by regressing the data to the Michaelis–Menten equation using GraFit Version 7.0.3.

### Assaying of hydrolytic activity

For assaying of β-galactosidase activity, reaction mixtures comprising 82.6 μg/ml IaSGT and 1 mM *p*NP-β-d-galactopyranoside in 20 mM sodium acetate buffer (pH 5.0) were incubated at 37 °C for 1 h. An equal volume of a 0.5 M Na_2_CO_3_ solution was added to each reaction mixture, and then the absorbance at 405 nm of each sample was measured. The extinction coefficient of pNP used was 18,500 M^−1^ cm^−1^. In order to evaluate the hydrolytic activity toward Sop_3_, a reaction mixture comprising 82.6 μg/ml IaSGT and 20 mM Sop_3_ in 20 mM sodium acetate buffer (pH 5.0) was incubated at 30 °C for 1 h. The concentrations of Glc released from Sop_3_ were determined by the GOPOD method described above. All experiments were carried out in triplicate.

### Crystallization, data collection, structure solution, and refinement

The screening of crystallization conditions was performed by the sitting-drop vapor diffusion method using Wizard Classic 1&2 (Emerald Biosystems), PEG/Ion Screen (Hampton Research), and Crystal Screen HR2-110 (Hampton Research). A reservoir solution (1 μl) and 3.5 mg/ml IaSGT solution (1 μl) were mixed and then incubated at 25 °C on 96-wells CrystalQuick plates (Greiner Bio-One) with the corresponding reservoir solution (70 μl). The protein crystals were grown in 200 mM calcium acetate, 100 mM Tris-HCl (pH 7.5), and 20% (w/v) PEG3350. Finally, the best diffracting crystals were obtained in the presence of 200 mM calcium acetate, 100 mM Tris-HCl (pH 7.5), and 12.5% (w/v) PEG3,000. Before X-ray data collection, protein crystals were transferred to reservoir solutions supplemented with 20% PEG400 as a cryoprotectant. If needed, 20 mM ligands were added to the solutions. The crystals were kept at 100 K under a nitrogen gas stream during data collection. The X-ray diffraction data were collected using a CCD detector (ADSC Q210) on beamline BL-5A and NW-12A at the Photon Factory. The diffraction data sets were processed using X-ray Detector Software (http://xds.mpimf-heidelberg.mpg.de/) (56). The initial phase of the selenomethionine-labeled IaSGT structure was determined by the single-wavelength anomalous dispersion method using AutoSol in Phenix (57). Automated model building was performed with Buccaneer (58). Molecular replacement was performed using MOLREP (59) to determine the initial phases of other structures (https://www.ccp4.ac.uk). Automated and manual structure refinements were performed using Refmac5 (60) and Coot (61), respectively (https://www.ccp4.ac.uk). The figures were drawn using PyMOL (http://www.pymol.org).

## Data availability

All relevant data are within the manuscript and its supporting information. The atomic coordinates and structure factors (codes 7VKW, 7VKX, 7VKY, 7VKZ, 7VL0, 7VL1, 7VL2, 7VL3, 7VL4, 7VL5, 7VL6, and 7VL7) have been deposited in the PDB.

## Supporting information

This article contains supporting information (52, 63, 64, 65, 66).

## Conflict of interest

The authors declare that there is no conflict of interest with the contents of this article.

## Supporting information

Supplemental Figures S1–S12 and Tables S1–S3

Supplemental Figures S2B
