# Supplementary material for: Characterization and structural analyses of a novel glycosyltransferase acting on the β-1,2-glucosidic linkages
Source: J Biol Chem. 2022 Jan 19;298(3):101606. doi: 10.1016/j.jbc.2022.101606 (PMC8861115; doi:10.1016/j.jbc.2022.101606)
Supplement: Supplemental Figures S1–S12 and Tables S1–S3 [file mmc1.docx]

SUPPORTING INFORMATION

**Characterization and structural analyses of a novel glycosyltransferase acting on the β-1,2-glucosidic linkages**

Authors

Kaito Kobayashi, Hisaka Shimizu, Nobukiyo Tanaka, Kouji Kuramochi, Hiroyuki Nakai, Masahiro Nakajima*, Hayao Taguchi

* To whom correspondence should be addressed: Department of Applied Biological Science, Faculty of Science and Technology, Tokyo University of Science, 2641 Yamazaki, Noda, Chiba 278-8510, Japan; m-nakajima@rs.tus.ac.jp

Contents:

**Supplementary Table S1**: A list of the amino acid sequences used in Figure S2.

**Supplementary Table S2**: Crystallographic statistics.

**Supplementary Table S3**: A list of the primers used in this study.

**Supplementary Figure S1**: Gene cluster of GH144 genes in *I. album*.

**Supplementary Figure S2**: Phylogenetic tree of IaSGT with GH35 enzymes.

**Supplementary Figure S3**: Alignment of IaSGT and TkGlmA.

**Supplementary Figure S4**: SDS-PAGE (A) and size-exclusion chromatography (B).

**Supplementary Figure S5**: pH (A) and temperature (B) profiles.

**Supplementary Figure S6**: ^1^H-NMR analysis of a glycosyltransfer product.

**Supplementary Figure S7**: Electrospray ionization-mass spectrometry analysis of the reaction products.

**Supplementary Figure S8**: Glycosynthase activity toward various glucosides.

**Supplementary Figure S9**: Binding mode of DNJ molecules.

**Supplementary Figure S10**: Multiple alignment of IaSGT homologs.

**Supplementary Figure S11**: Comparison of substrate recognition at subsite −1 between the IaSGT WT-Sop_2_ (A), TkGlmA-GlcN (B), and SpBgaC-Gal (C) complexes.

**Supplementary Figure S12**: Speculated physiological role of IaSGT.

**Table S3. A list of the primers used in this study.**

| Cloning of IaSGT (5′-oligonucleotide-3′) | | |
| --- | --- | --- |
|  | Forward | Reverse |
|  | GAATTTAAACAATCATATGAAGCCGCATAATTCTG | TAAAAAGTTCTCGAGTTTGTTCTCTTTTATAAC |
| Mutation of IaSGT (5′-oligonucleotide-3′) | | |
| Mutant | Forward | Reverse |
| E176Q | CTGCAATCAAATTGGTGTGTTTCAATG | CCAATTTGATTGCAGAGCTGCATCATT |
| E343Q | GGCTGCACAGTTTCAGGCAGGAACAAG | TGAAACTGTGCAGCCCAAACAGGAGCT |
| E343G | GGCTGCAGGGTTTCAGGCAGGAACAAG | TGAAACCCTGCAGCCCAAACAGGAGCT |

**Figure S1. Gene cluster of GH144 genes in *I. album*.**

Genes encoding putative glycoside hydrolases and related to transporters are shown as blue and green arrows. The gene encoding IaSGT is shown in red. Gray arrows indicate that prediction of functions is difficult. Numbers in arrows are locus numbers based on the KEGG database (https://www.genome.jp/kegg/).

**Figure S2. Phylogenetic tree of IaSGT with GH35 enzymes.**

(A) The amino acid sequences were aligned using MEGA X (1) and the tree was constructed using the likelihood method. A bacterial group, two eucaryotic groups and an archaeal group are represented by dashed lines. IaSGT is indicated by a red spot. Structurally available enzymes are indicated by blue spots, PDB IDs, and organism names. PDB IDs of the structures used in Fig. S11BC are shown by blue bold letters. The *scale bar* represents the number of substitutions per amino acid site. A list of the amino acid sequences used in the figure is given in Table S1. Structurally available enzymes in the archaeal group and enzymes in the other groups except IaSGT homologs are all characterized enzymes. (B) The same tree with the accession numbers and the organism names is provided as a separate file.

**Figure S3**. **Alignment of IaSGT and TkGlmA.**

Alignment was performed by ClustalW2 (https://www.ebi.ac.uk/Tools/msa/clustalw2/) (2) and the figure was prepared with the Espript 3.0 server (https://espript.ibcp.fr) (3). Conserved recognition residues of GlcN and catalytic residues in TkGlmA are indicated by black and pink ovals, respectively. The GlcN recognition residue indicated by a blue oval is not conserved in IaSGT.

**Figure S4. SDS-PAGE (A) and size-exclusion chromatography (B).**

(A) A 12% polyacrylamide gel was used. M, a mixture of standard proteins (Precision plus protein standards) purchased from Bio-rad (CA, USA) was loaded on the gel; IaSGT, the purified recombinant IaSGT. (B) Standard proteins and IaSGT are shown as closed circles and an open square.

**Figure S5. pH (A) and temperature (B) profiles.**

(*left*) Optimum activity and (*right*) residual activity. Median values of triplicate experiments are plotted as closed symbols and the other values are shown as both edges of error bars. (A, *left*) Closed circles and triangles represent Briton-Robinson buffer and sodium acetate buffer, respectively.

**Figure S6. ^1^H-NMR analysis of a glycosyltransfer product produced by IaSGT.**

(A) Purification of the reaction product from Sop_3_ as a starting material using a Toyopearl HW-40F column. Lane M, a marker containing each 0.5% Glc and Sop_2−5_; lane 1−8, fraction numbers of collected fractions. At each lane, 1 μl solution was spotted. Only fraction 7 was used for NMR analysis. (B) A glycosyltransfer product, (C) reference Sop_4_. The ratio of integrals of H-2, 3, 4, 5, 6 and 6’ (all Glc moieties) to that of H-1 (sum of both anomers at the reducing end) are 17.02 and 17.00 for (B) and (C), respectively. The difference in anomer ratios between the two samples is probably due to the amounts of acetone added to the samples as an internal standard.

**Figure S7. Electrospray ionization-mass spectrometry analysis of the reaction products.**

Basically, the peaks are assigned as [M + NH_4_]^+^ and shown by red and blue arrows for the products and the substrates, respectively. Other types of ions are shown above the peaks by red dashed lines. (A) The reaction products from Sop_5_ (10 mM). The numbers above the arrows and dashed lines are DPs of the products. The letters “DP” are omitted except DP5. The bottom spectrum is the same one at the middle except for the scale. (B) The reaction products from phenyl-α-Glc (10 mM) and Sop_2_ (10 mM). The compounds related to the reaction are represented schematically with white circles and gray hexagons for Glc and phenol moieties, respectively.

**Figure S8. Glycosynthase activity toward various glucosides.**

The presence and absence of the E343G mutant in the reaction mixtures are shown as + and –. Acceptors are shown below the TLC plates. Since α-GlcF acted as an acceptor, the positions of α-GlcF and putative Glc-GlcF are given at the left of the plates. The parentheses indicate that the product was not identified. The arrow at the right of the plate indicates the reaction product derived from pNP-α-Glc.

**Figure S9. Binding mode of DNJ molecules.**

DNJ molecules were observed only in subunit A. The representation of the figure follows that in Fig. 6. The Sop_2_ molecule in the WT-Sop_2_ complex is superimposed.

**Figure S10. Multiple alignment of IaSGT homologs.**

IaSGT homologs were searched for in the KEGG website and the top 13 were used for figure preparation using Espript 3.0 as in Fig. S3. They are from *Anoxybacter fermentans* (locus number, BBF96_10410), *Halobacteroides halobios* (Halha_0234), *Cohnella candidum* (EAV92_21545), *Sulfobacillus acidophilus* DSM10332 (Sulac_2024), *Sulfobacillus acidophilus* TPY (TPY_1821), *Sulfobacillus thermotolerans* (BXT84_05270), *Fictibacillus arsenicus* (ABE41_017750), *Fictibacillus phosphorivorans* (ABE65_018505), *Sediminibacillus* sp. DP4-553-S (ERJ70_01935), *Halobacillus mangrovi* (HM131_05715), *Tenericutes bacterium* MZ-XQ (BK011_09420), *Mesotoga prima* (Theba_2484), and *Fervidobacterium pennivorans* (Ferpe_0817). Substrate recognition residues at subsites −1 and +1 with hydrogen bonds with main and side chains except Arg349 are indicated by black ovals and triangles, respectively. Leu100 and Arg349 are indicated by red stars. The hydrophobic pocket residues in helix α6 are indicated by blue ovals. Black reverse triangles represent the residues involved in recognition at subsite +2 by side chains. Catalytic residues are indicated by pink ovals. The potential recognition residue of O6 atom with *tg* rotamer is indicated by white oval.

**Figure S11. Comparison of substrate recognition at subsite −1 between the IaSGT WT-Sop_2_ (A), TkGlmA-GlcN (B), and SpBgaC-Gal (C) complexes.**

Residues considered to be involved in substrate specificity are shown as thicker sticks with bold residue names. The other residues are shown as thinner sticks. Ligands at subsite −1 are shown as thicker sticks. (Glc) represents the Glc moiety in Sop_2_. Residues and ligands in IaSGT, TkGlmA and SpBgaC are shown as green and white, light pink and yellow sticks, and cyan and light blue sticks, respectively. Hydrogen bonds are shown as blue dashed lines. The orientation of O4 atoms and the rotamer of O6 atoms are given in parentheses. The PDB IDs used are 5GSM and 4E8C for the TkGlmA-GlcN and SpBgaC-Gal complexes, respectively.

**Figure S12. Speculated physiological role of IaSGT.**

The speculated roles of the proteins encoded in the gene cluster (see Fig. S1) in the *I. album* are illustrated. The locations of proteins shown in the figure are based on prediction with the Gneg-mPLoc server (http://www.csbio.sjtu.edu.cn/bioinf/Gneg-multi/) (4). Since the two putative SGLs and a putative SOGP have no transmembrane region, they may be peripheral membrane proteins. The rather high possibility of N-terminal signal peptides in the SGLs according to prediction with the SignalP server (http://www.cbs.dtu.dk/services/SignalP/) (5) implies localization on the periplasm side. The putative SOGP with no signal peptide is expected to be located on the cytoplasm side. The reaction in this study and the predicted reactions based on annotations are represented as solid and dashed lines. The transportation by the TonB-dependent transporter is shown as a thin dotted line due to lack of biochemical evidence for prediction.

**References**

1. Kumar, S., Stecher, G., Li, M., Knyaz, C., and Tamura, K. (2018) MEGA X: Molecular evolutionary genetics analysis across computing platforms. *Mol. Biol. Evol.* **35**, 1547–1549

2 Larkin, M. A., Blackshields, G., Brown, N. P., Chenna, R., Mcgettigan, P. A., McWilliam, H., Valentin, F., Wallace, I. M., Wilm, A., Lopez, R., Thompson, J. D., Gibson, T. J., and Higgins, D. G. (2007) Clustal W and Clustal X version 2.0. *Bioinformatics*. **23**, 2947–2948

3. Robert, X., and Gouet, P. (2014) Deciphering key features in protein structures with the new ENDscript server. *Nucleic Acids Res.* **42**, 320–324

4. Shen, H.B., and Chou, K.C. (2010) Gneg-mPLoc: A top-down strategy to enhance the quality of predicting subcellular localization of Gram-negative bacterial proteins. *J. Theor. Biol.* **264**, 326–333

5. Almagro Armenteros, J. J., Tsirigos, K. D., Sønderby, C. K., Petersen, T. N., Winther, O., Brunak, S., von Heijne, G., and Nielsen, H. (2019) SignalP 5.0 improves signal peptide predictions using deep neural networks. *Nat. Biotechnol.* **37**, 420–423
