# Supplementary figures and images for: Characterization and structural analyses of a novel glycosyltransferase acting on the β-1,2-glucosidic linkages
Source: J Biol Chem. 2022 Jan 19;298(3):101606. doi: 10.1016/j.jbc.2022.101606 (PMC8861115; doi:10.1016/j.jbc.2022.101606)

Archaeal  
group

## Eukaryotic

Eukaryotic  
group 2

Eukaryotic  
group 1

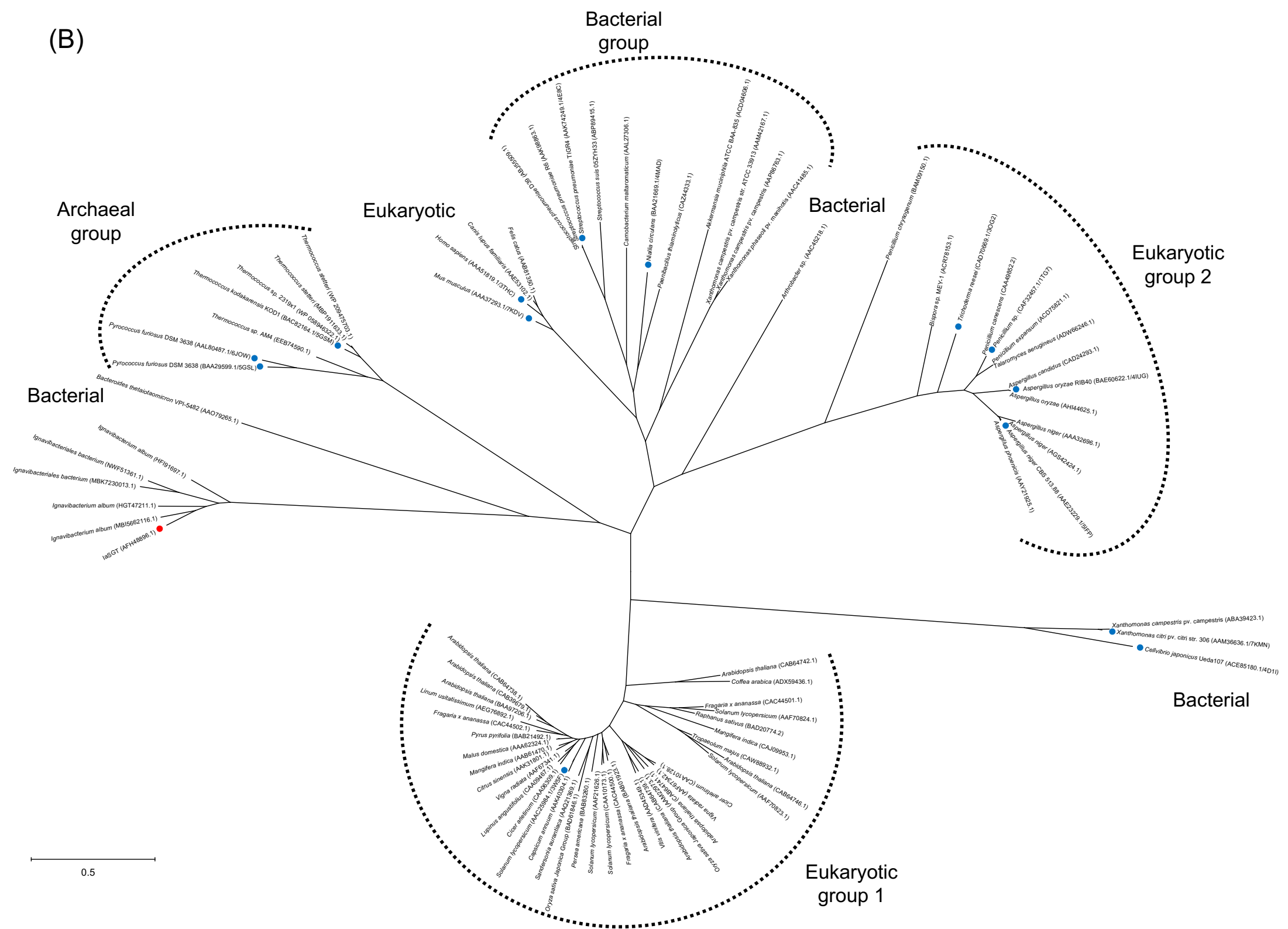

Supplement: Supplemental Figures S2B [file mmc2.pdf]
